# Characterization and genomic analyses of two newly isolated *Morganella* phages define distant members among *Tevenvirinae* and *Autographivirinae* subfamilies

**DOI:** 10.1038/srep46157

**Published:** 2017-04-07

**Authors:** Hugo Oliveira, Graça Pinto, Ana Oliveira, Jean-Paul Noben, Hanne Hendrix, Rob Lavigne, Małgorzata Łobocka, Andrew M. Kropinski, Joana Azeredo

**Affiliations:** 1 CEB – Centre of Biological Engineering, LIBRO – Laboratório de Investigação em Biofilmes Rosário Oliveira, University of Minho, 4710-057 Braga, Portugal; 2 Biomedical Research Institute and Transnational University Limburg, Hasselt University, Diepenbeek 3590, Belgium; 3 Laboratory of Gene Technology, KU Leuven, Kasteelpark Arenberg 21 box 2462, B-3001 Leuven, Belgium; 4 Department of Microbial Biochemistry, Institute of Biochemistry and Biophysics of the Polish Academy of Sciences, Warsaw, Poland; 5 Autonomous Department of Microbial Biology, Faculty of Agriculture and Biology, Warsaw University of Life Sciences, Warsaw, Poland; 6 Departments of Food Science; Molecular and Cellular Biology; and, Pathobiology, University of Guelph, Guelph, ON N1G 2W1, Canada

## Abstract

*Morganella morganii* is a common but frequent neglected environmental opportunistic pathogen which can cause deadly nosocomial infections. The increased number of multidrug-resistant *M. morganii* isolates motivates the search for alternative and effective antibacterials. We have isolated two novel obligatorily lytic *M. morganii* bacteriophages (vB_MmoM_MP1, vB_MmoP_MP2) and characterized them with respect to specificity, morphology, genome organization and phylogenetic relationships. MP1’s dsDNA genome consists of 163,095 bp and encodes 271 proteins, exhibiting low DNA (<40%) and protein (<70%) homology to other members of the *Tevenvirinae*. Its unique property is a >10 kb chromosomal inversion that encompass the baseplate assembly and head outer capsid synthesis genes when compared to other T-even bacteriophages. MP2 has a dsDNA molecule with 39,394 bp and encodes 55 proteins, presenting significant genomic (70%) and proteomic identity (86%) but only to *Morganella* bacteriophage MmP1. MP1 and MP2 are then novel members of *Tevenvirinae* and *Autographivirinae*, respectively, but differ significantly from other tailed bacteriophages of these subfamilies to warrant proposing new genera. Both bacteriophages together could propagate in 23 of 27 *M. morganii* clinical isolates of different origin and antibiotic resistance profiles, making them suitable for further studies on a development of bacteriophage cocktail for potential therapeutic applications.

Bacteriophages (phages) are bacterial viruses which have received renewed interest as alternative or complementary antibacterial agents in the fight against multidrug-resistant pathogens^1^. Phages are also important genetic tools that have contributed significantly to the development of the fields of molecular biology and biotechnology^2^,^3^.

Phage taxonomy was originally based on phage morphology and genome type. Phages of complex virions which consist of a head that contain linear dsDNA and a tail have been classified to a separate order - *Caudovirales* (tailed phages)^4^. They evolved before the separation of bacteria and archaea. Their long evolutionary history, as well as, frequent genetic exchanges of their genes by horizontal gene transfer has led to the enormous diversification of this group of viruses and often the disappearance of sequence similarities between members of the same families, at the DNA or protein level. Thus, uniform rules for classifying tailed phages, analogous to those, based on the sequence of the 16S rRNA gene that are used for bacteria, do not exist. For example, the *Salmonella* virus P22 and the coliphage T7 have been placed in the same *Podoviridae* family on the basis of morphology despite the lack of DNA sequence relatedness between them^5^. With the advance of genomics, newer approaches based on the comparative analysis of terminase sequences^6^, structural genes^7^ or whole proteomes^8^ have been proposed to classify phages. More recently, a novel BLAST-based function prediction has emerged as a novel tool to distinguish phage subfamilies^9^. Therefore, the taxonomic positioning of phages is in constant evolution due to the continuous deposition of a large number of novel genomes in the GenBank, especially for phages targeting clinically relevant pathogens.

*Morganella morganii* is an opportunistic pathogen often associated with nosocomial infections^10^, with several clinical isolates being resistant to multiple antibiotics^11^. Several studies have demonstrated the frequency of spontaneous antibiotic resistance (e.g. β-lactams)^12^,^13^,^14^, with the ability to acquire new resistance mechanisms (e.g. horizontal transfer of resistance genes)^15^,^16^. Relative to other Gram-negative bacteria such as *Escherichia coli* and *Pseudomonas aeruginosa*, only a few phages have been characterized against *M. morganii*. Historically, several phage typing systems were developed for this bacterium^17^,^18^,^19^,^20^ and a generalized transducing phage, subsequently called phage M^21^,^22^,^23^, has also been reported. More recently, *Morganella* phage FSP1 was characterized as a member of the *Myoviridae* with a genome of approximately 47.7 kb^24^. To our knowledge, only one *Morganella* phage has been sequenced, but is lacking an associated microbiological characterization (phage MmP1)^25^.

This study reports the isolation and characterization of two novel phages that are able to propagate in multidrug resistant *M. morganii* isolates, and may potentially find a therapeutic use. They are named MP1 (vB_MmoM_MP1) and MP2 (vB_MmoP_MP2), according to recent nomenclature recommendations^26^. Morphological analyses and whole-genome comparisons reveal that MP1 and MP2 belong to *Myoviridae* and *Podoviridae* families and represent new clades within the *Tevenvirinae* and *Autographivirinae* subfamilies, respectively. This work provides a detailed characterization of MP1 and MP2 contributing to a better understanding of the biodiversity of phages infecting different hosts.

## Materials and Methods

### Bacterial strains and culture conditions

All bacterial strains used to evaluate the lytic spectra of the isolated phages have been listed in Table S1. The panel includes strains from the Salmonella Genetic Stock Centre, the American Type Culture Collection as well as several clinical isolates: 27 *M. morganii* and one representative of each of the following closely-related species: *Citrobacter freundii* and *Citrobacter koseri, Proteus mirabilis, Proteus vulgaris, Providencia stuartii* and *E. coli*. Clinical isolates have undergone typing and antimicrobial susceptibility tests with Vitek2 (BioMerieux) or Walk Away (Beckman Coulter), according to CLSI guidelines. Strains producing beta-lactamases (AmpC) were identified using an Etest (BioMerieux) with positive results for cefotetan/cefotetan + cloxacillin. Bacteria were grown at 37 °C in Tryptic Soy Broth (TSB, Merck) or in Tryptic Soy Agar (TSA, Merck).

### Phage Isolation and propagation

Phages were isolated from wastewater treatment plant sewage samples located in Braga (Portugal) following the enrichment procedure described previously^27^. Briefly, 20 ml of 2x TSB were added to 20 ml of previously centrifuged (10 min, 9,000 × *g*, 4 °C) raw sewage. After that, a loop of each *M. morganii* bacterial strain was added, and the mixture was incubated for 16 h (37 °C, 120 rpm, ES-20/60). The suspension was centrifuged (10 min, 9,000 × *g*, 4 °C) and the presence of phages in the supernatant was confirmed by spotting 10 μl on each of the bacterial lawns, previously incorporated in 0.6% TSA. After a 16 h incubation period at 37 °C, clear and turbid areas on the lawns were considered positive for phages and single plaques were repeatedly purified until only one plaque morphology was achieved.

Phages were propagated in accordance with a previously described protocol^27^. Briefly, purified phages were incorporated into a 0.6% TSA layer with bacteria, and incubated at 37 °C for 16 h. SM buffer (5.8 g/L NaCl, 2 g/L MgSO_4_.7H_2_O, 50 mL 1 M Tris, pH 7.5) was added to the plates, which were then incubated at 4 °C (16 h, 120 rpm, ES-20/60). After that, all liquid was removed from the Petri dishes, and 0.58 g of NaCl was dissolved in each 10 mL of sample. The resultant suspension was incubated for 1 h at 4 °C, centrifuged for 10 min (9,000 × g, 4 °C), and the supernatant was collected. Phages were concentrated via PEG 6000 precipitation and purified with chloroform as described previously^28^. Briefly, PEG 6000 (10% (w/v)) was added to the samples and incubated for 16 h at 4 °C. The pellet (containing phages) was recovered by centrifugation (10 min, 9,000 × *g*, 4 °C), suspended in SM buffer and purified with chloroform (1:4 (v/v)). After centrifugation (15 min, 3,000 × *g*, 4 °C), the aqueous phase was collected, filtered through a 0.22 μm Whatman PES membrane and stored at 4 °C.

### Lytic spectra and efficiency of plating (EOP)

The lytic spectra of phages were evaluated using the drop spot test. Phages were tested against all the bacterial strains listed in Table S1. The phage titers were adjusted to 10^8^ PFU/mL, 10-fold serial diluted, and 10 μL of each dilution were spotted onto the lawns of each strain, before incubation for 16 h at 37 °C. The results were observed and a score attributed. The relative efficiency of plating (EOP) was calculated by dividing the titer of the phage (PFU/mL) of each isolate by the titer for the relevant propagating host.

### One-Step-Growth curve

One-step growth curves were performed as described previously^2^. Briefly, 10 ml of a mid-exponential-phase culture were harvested by centrifugation (9,000 × *g*, 5 min, 4 °C) and suspended in 5 ml of fresh TSB to an OD_620_ of 1.0. An equal volume of phage was added in order to have a multiplicity of infection (MOI) of 0.001. This culture was then incubated at room temperature for 5 min to allow phage adsorption to the host. The mixture was centrifuged as described above, the pellet was suspended in 10 ml of fresh TSB medium and incubated at 37 °C. Samples were collected for plaque forming unit counts at 5, 10, 15, 20, 25, 30, 40, 50 and 60 min and plated immediately. Averages ± standard deviations for all experiments are given for n = 3 repeats.

### Cytotoxicity assay

The cell line A549 (ATCC CCL-185) was grown in Dulbecco’s Modified Eagle’s Medium (DMEM with 3.7 g/L NaHCO_3_ and 4.5 g/L D-glucose, Biochrom) supplemented with 10% of fetal bovine serum (FBS, Biochrom) and 1% Penicillin/Streptomycin (Sigma-Aldrich), at 37 °C in a humidified incubator with 5% CO_2_. Cells were seeded into wells of a 96-well plate at 1 × 10^6^ cells/mL and incubated for 24 h, to reach a cell confluence between 70 and 90%. Phage solution (in SM buffer) was added to the cells, so that a phage concentration of 10^8^ PFU/mL and a final volume of 100 μL were achieved in the wells. Controls were performed by adding the same amount of SM buffer to the wells with cells grown in the same conditions. After 24 h, 20 μL of CellTiter 96 Aqueous One Solution Reagent (Promega) was added to each well and incubated for 1 h at 37 °C in a humidified 5% CO_2_ atmosphere, protected from light. The amount of soluble formazan produced by cellular reduction of MTS was measured by reading the absorbance at 490 nm. The experiment was performed in triplicate.

### Mass spectrometry

A protein pellet from the phage virion proteins was obtained after a chloroform:methanol extraction (1:1:0.75, v/v/v) performed for both phage on high titre of purified samples (>10^10^ PFU/mL). Subsequently, this pellet was resuspended in loading buffer (1% SDS, 6% sucrose, 100 mM dithiothreitol, 10 mM Tris pH 6.8, 0.0625% w/v bromophenol blue) and heated to 95˚C (5 min). Samples were run on a 12% SDS-PAGE gel and stained using Gelcode™ Blue Safe Protein Stain (Thermo Scienitic, Waltham, Massachusetts, United States) to visualise virion proteins. Gel fragments were extracted and subjected to trypsinization for analysis using tandem electrospray ionization-mass spectrometry (ESI-MS/MS) exactly as described previously^29^.

### Transmission electron microscopy

Phage particles were sedimented (20,000 × *g*, 1 h, 4 °C), using a Beckman J2-21 (Carlsbad, CA, USA) centrifuge, washed twice and resuspended in tap water. Phages were deposited on copper grids provided with carbon-coated Formvar films, stained with 2% uranyl acetate (pH 4.0), and examined using a Jeol JEM 1400 transmission electron microscope.

### Genome sequencing and assembly

Phage genomic DNA was isolated using the proteinase K and sodium dodecyl sulphate, followed by phenol:chloroform extraction as described previously^28^. DNA was sequenced by the VIB Nucleomics Core (Leuven, Belgium) using a NGS Illumina MiSeq platform (Illumina Inc., San-Diego, USA) and a NEBNext^®^ Ultra™ DNA Library Prep Kit to generate 500-bp fragments with 2 × 300 bp paired-end read length configuration. Quality controls of DNA before being sequenced on the MiSeq System were made by Agilent Bioanalyzer and Qubit measurements. Outputs were demultiplexed and *de novo* assembled into a single contig using CLC Bio Genomics Workbench v7.0 (Aarhus, Denmark) and manually inspected. To identify virion DNA ends the assembly patterns of subpopulations of random sequence reads representing the genome fragments of each phage (up to 100x coverage) were analysed using the SeqMan program of Lasergene 9.1 package (DNAStar, Madison, WI, USA).

### Genome analysis

The phage’s genome was annotated using MyRAST^30^. The CDSs putative functions were assigned by BLASTP^31^ queried against non-redundant protein sequences databases. HHpred^32^ was used for the detection of homology and structure prediction. Genes coding tRNAs were predicted using tRNAscan-SE^33^ and ARAGORN^34^. Putative promoters were identified using 100 bp of CDS upstream DNA sequence and MEME^35^, followed by manual confirmation. The putative rho-independent terminators were identified using ARNold^36^ and the free energy of their secondary structures was calculated using Mfold^37^. The transmembrane domains and signal peptides were found using Phobius^38^, TMHMM^39^, HMMTOP^40^ and SignalP^41^. The complete genome sequence of phages MP1 and MP2 have been deposited in the NCBI database under accession no. KX078569 and KX078568, respectively.

### Comparative analysis of the phage genomes

For MP1 the comparisons were made with *Proteus* phage vB_PmiM_Pm5461 (NC_028762), *Escherichia* phages T4 (NC_000866), R49 (NC_005066), RB69 (AY303349), CC31 (GU323318), JS98 (EF469154), *Shigella* phage SP18 (GQ981382), *Salmonella* phage S16 (NC_020416) and *Vibrio* phage KVP40 (NC_005083). MP2 was compared with *Morganella* phage MmP1 (NC_011085), *Escherichia* phage T7 (NC_001604), *Salmonella* phage SP6 (NC_004831), *Pseudomonas* phages φKMV (AJ505558) and *Klebsiella* phage KP34 (GQ413938). At the DNA level, homology comparisons between phages were conducted with progressiveMauve^42^. Dot plots of the whole-genomes were produced using Gepard under default parameters^43^. At the proteomic level, CoreGenes^44^ was used to analyze the proteome conservation between phages. Pairwise comparisons were also made using tbBLASTX plug-in in Easyfig^45^ to reveal intercluster relationships. The phylogenetic analysis of homologous phage large terminase subunit or DNA polymerase proteins was conducted in “one click” mode at phylogeny.fr (http://www.phylogeny.fr/)^46^. The trees were exported in Netwick format and visualized using FigTree (http://tree.bio.ed.ac.uk/software/figtree/).

## Results and Discussion

### Microbiological characterization of the *Morganella* phages

MP1 and MP2 were isolated from wastewater treatment plant sewage samples using *Morganella* strains from different human clinical specimens in the enrichment. The phages’ lytic spectra were assessed against a wide range of clinical isolates and six strains of other closely-related *Enterobacteriaceae* species (Table S1). MP1 and MP2 were able to lyse 67% and 56% of all the *Morganella* isolates tested, respectively. Therefore, *in vitro* assessments revealed their infection patterns to be promising for therapy. In addition, they have a complementary lytic range infecting a total of 85% of the strains, and showed to be specific, as they were unable to lyse the remaining *Enterobacteriaceae* species tested. Their ability to kill drug resistant *M. morganii* clinical isolates (AmpC strains, resistant up to 11 antibiotics) makes them especially promising antibacterials of potential use against hard-to-treat infections by *M. morganii* strains. The latent periods and burst sizes were also determined based on the results of one-step growth experiments. Phage MP2 bursts after 20 min releasing only 16 progeny viruses per cell, while MP1 has a longer (35-min) latent period and also higher burst size (41 phages) properties which it shares with *Morganella* phage FSP1^24^.

To assess the potential safe application of these phages, cytotoxicity tests were performed. Since *M. morganii* is known to cause several nosocomial infections, including pneumonia and urinary tract infections, MP1 and MP2 cytotoxic effect was evaluated using the cell line A549 (ATCC CCL-185), derived from a human alveolar cell carcinoma^47^. Similar quantities of soluble formazan detected from A549 cells after 24 hours exposure to MP1 or MP2, compared with the SM buffer, are indicative of absence of toxicity (Figure S1). As such, no counter indications towards the application of MP1 and MP2 to treat bacterial infections *in vivo* have been observed.

### MP1 and MP2 morphologies

**Figure 1:**
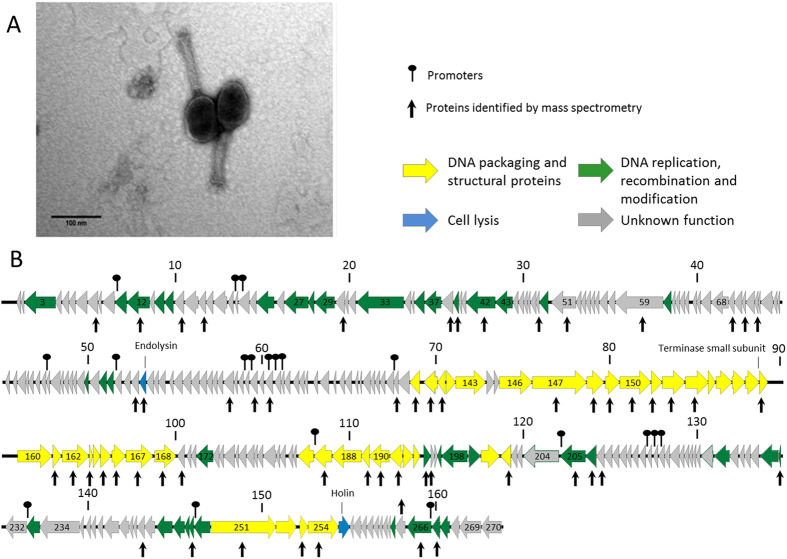
Morphology and genome overview of MP1. (**A**) Electron micrographs of *Morganella*-infecting phage MP1 negatively stained with 2% uranyl acetate. (**B**) Genome map with predicted 271 CDSs numbered and coloured (yellow, green, blue and gray) according to their predicted function. Protein identified by mass spectrometry are pointed with vertical arrows. Above genome, the nucleotide positions in kb are given.

**Figure 2:**
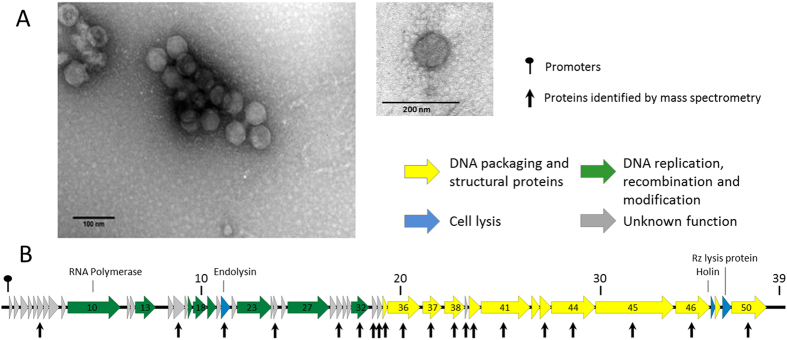
Morphology and genome overview of MP2. (**A**) Electron micrograph of *Morganella*-infecting phage MP2 negatively stained with 2% uranyl acetate. (**B**) Genome map with predicted 55 CDSs numbered and coloured (yellow, green, blue and gray) according to their predicted function. Protein identified by mass spectrometry are pointed with vertical arrows. Above genome, the nucleotide positions in kb are given.

MP1 produces uniformly small plaques (0.1-mm diameter), which are difficult to enumerate even in a bacterial lawn solidified with 0.4% agar. MP2 always forms small (0.5-mm diameter) clear and large (2-mm diameter) plaque variants, both surrounded by haloes for each *Morganella* strain tested. The presence of opaque halos suggests active production of exopolysaccharide depolymerase-like enzymes^48^, which have never been described for *Morganella* phages. Transmission electron micrographs revealed two morphotypes (Figs 1A and 2A). MP1 is a myovirus with an elongated icosahedral head (85 nm in length and 65 nm in width) and a contractile tail of 111 nm long and 17 nm wide. MP2 has a smaller isometric head between 48 and 51 nm in diameter and a short (7 nm) non-contractile tail, which is a feature common to members of the *Podoviridae*^4^. MP1 and MP2 are then morphologically similar to *Morganella* phages FSP1 and MmP1, respectively.

### Genome general characteristics

The MP1 and MP2 genomes are both linear dsDNA molecules of 163,095 bp and 39,394 bp, respectively. The MP1 genome has a GC content of 34.7% whereas MP2 genome GC content is 46.9%. These values fall below the GC content of 51% observed in sequenced *M. morganii* strains^49^,^50^. The assembly pattern of MP1 sequence reads indicated a terminal redundancy and a circular permutation of virion DNA molecules, as observed for coliphage T4, whose DNA is packed into capsids from concatamers, by a headful mechanism^51^. The assembly pattern of MP2 sequence reads is characteristic for phages with a sequence specific packaging site and indicated the presence of 200-bp terminal repeats at ends of its genome, similar to that of coliphage T7^52^. Genes in both, MP1 and MP2, are densely packed and occupy 95% and 89% of DNA, respectively. The genes mostly belong to one of the three distinct functional modules: DNA packaging and structural proteins; DNA replication, recombination and modification; and cell lysis (Figs 1B and 2B). While in the case of MP2 the genes of particular modules occupy distinct genome regions, in MP1 they are dispersed throughout the genome.

MP1 DNA encodes 271 predicted proteins with coding sequences situated on both the positive and negative strands and 10 tRNAs. The translation of 263 of these proteins starts from an ATG codon, five from a TTG codon, and three from a GTG codon. For 109 of the MP1 proteins a specific function can be assigned, while the other 162 have no known function, including 86 which are unique to this phage (Table S2). Identified homologs of MP1 proteins are mostly encoded by *Proteus* phage vB_PmiM_Pm5461. Proteins of assigned function include several structural proteins such as the major capsid, tail and neck proteins and proteins related to DNA replication, packaging and regulation. Genes encoding a small and large subunit of aerobic ribonucleotide reductase are separated from each other by a gene encoding HNH-type homing endonuclease (*gp233*), similarly as it is in the case of Enterobacteria phages RB32 and T4. A few proteins show similarities to products of bacterial genes (e.g. *M. morganii, Xenorhabdus griffiniae, Escherichia coli*). One example is the endolysin gene (*gp100*), which encodes a protein with peptidase activity (VANY, PG02557) with similarity to an *E. coli* peptidoglycan hydrolase. Regarding regulatory elements, 12 promoters and 27 rho-independent terminators were identified.

MP2 has a modular genome containing 55 proteins coding genes, all transcribed in the Watson strand. The functions of 24 proteins can be predicted (Table S3). Most proteins are closely related to those of phage MmP1, with identities ranging from 53 to 100%. Only the products of two predicted genes are homologous to proteins from different phages, namely *Citrobacter* phage CR8 and *Escherichia* phage CICC. Genes of assigned function encode several structural proteins, such as the major capsid protein, the capsid assembly protein, several tail proteins (tail tubular protein A and B, tail fiber, head-to-tail joining protein) and DNA replication and transcription-associated proteins, DNA polymerase and RNA polymerase among them. It was possible to identify *gp21* (endolysin), *gp47* (holin) and *gp49* (Rz-like spanin) encoding the lytic cassette. The functions of the remaining 31 protein coding genes are unknown, being 16 of them unique to this phage. Only two host-dependent promoters, and three rho-independent terminators were identified in MP2. All genes start with the ATG codon, with the exception of *gp50* (DNA packaging protein), which starts with TTG.

### Phage structural proteins

**Figure 3:**
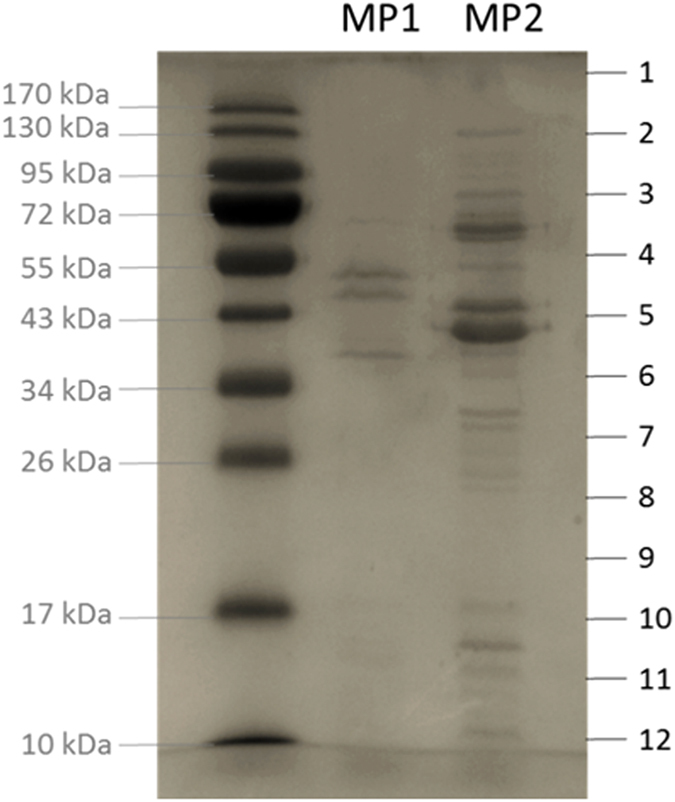
SDS-PAGE analysis of the purified structural proteins of MP1 and MP2. A 12% SDS-PAGE separation gel of phage proteins were made alongside with a PageRuler^TM^ prestained protein ladder. The entire lane was cut into 12 slices for mass spectrometry analysis.

To determine the protein composition of MP1 and MP2 virions, the structural components of both phages were precipitated, separated by thin-layer SDS-PAGE, trypsinized and analysed by electrospray ionization-tandem mass spectrometry (Fig. 3, Tables 1 and 2). Although some proteins were clearly visualized with Simply Blue™ Safe Stain, the whole gel was extracted, fragmented and analysed as previously described^53^. The resulting peptides allowed the identification of 58 and 21 proteins scattered around the MP1 and MP2 genome, respectively.

In MP1, 27 proteins had structural-related functions, being the baseplate wedge subunit (*gp147*), tail sheath (*gp162*), prohead core scaffold (*gp167*), major capsid (*gp168*) and the head vertex proteins, the most abundant (sequence coverage ranging from 37.9 and 79.9%). In several cases, however, the corresponding N- and C-terminal peptides were identified in several gel bands. This suggests posttranslational modifications, possibly due to proteolytic cleavage or degradation. Similar post-translational modifications have been previously observed in the case of T4 head proteins^54^. Furthermore, 20 proteins were identified with unknown functions, from which *gp7, gp18* and *gp73* that are located in the first halves of the MP1 genome, were unique. The remaining proteins identified specified unusual functions of DNA replication and cell lysis. Overall, for most identified proteins, relatively high homologies (>60% amino acid identity) were detected only to proteins of *Proteus* phage vB_PmiM_Pm546 (hereafter referred to as Pm5461).

In MP2, 12 proteins were previously predicted with structural function (*gp35*-*gp41, gp43*-*gp46, gp50*), including the internal virion protein, tail fiber and major capsid proteins. The product of *gp45* is likely to facilitate the passage of phage DNA through the cell wall at infection by introducing a small gap in the peptidoglycan layer. It contains the amino acid sequence motif characteristic for soluble lytic transglycosylases, similarly to virion proteins of certain other phages that have this activity^10^,^55^. Three additional proteins of unassigned functions, encoded by genes located in a close vicinity to each other were also detected and presumably, are also virion components (*gp33*-*gp34, gp39*). BLASTP showed that most of these proteins had a relatively high homology only to *Morganella* phage MmP1 (>60% amino acid identity). Furthermore, mass spectrometry allowed the detection of two unique proteins, *gp6* (22.6% coverage) and *gp29* (38.5% coverage), whose genes are located upstream and downstream of the predicted DNA replication module, respectively.

### Comparative genomics

**Figure 4:**
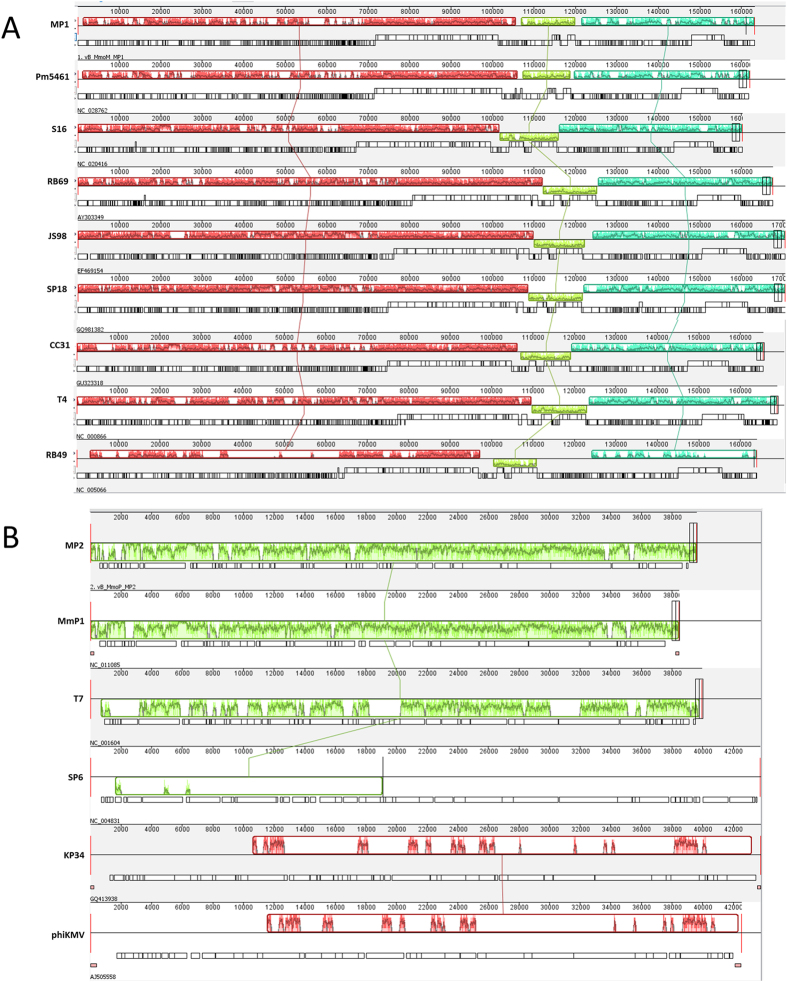
Multiple genome alignment of *Morganella* phage genomes against homologs. (**A**) MP1 and (**B**) MP2 were compared to *Tevenvirinae* and *Autographivirinae* like phages, respectively. Genomes were compared using Mauve software, and similarity profiles were generated. Boxes with identical colors represent local colinear blocks (LCB) indicating homologous DNA regions shared by two or more genomes, indicated by a connected line. The placement of a block below the axis indicates inversion. Similarity inside the block is indicated by the height of the colored bars. For ease of interpretation, myovirus *Vibro* phage KVP40 with a 244,834 bp size was excluded from the *Tevenvirinae* comparisons.

The Bacterial and Archaeal Viruses Subcommittee of the International Committee on Taxonomy of Viruses (ICTV) has taken a holistic approach to the classification of phages, employing overall DNA and protein sequence identities coupled with phylogenetic analyses^56^,^57^. An initial BLASTN analysis of the complete genome sequence of MP1 revealed that *Proteus* phage Pm5461 is its closest relative, but these phages only share 39% overall sequence identity. Phage MP2, only exhibits significant overall DNA sequence identity of 70% with *Morganella* phage MmP1, as the sequence identity decreased below 40% when compared to other phages. Therefore, the creation of novel genus containing only MP1 on one hand and grouping MP2 together with MmP1 on the other hand seems appropriate at this stage. Nevertheless, owing to low nucleotide homology found with MP1 and MP2 against other viruses, we decided to perform multiple genome alignments with all prototype viruses currently classified within *Tevenvirinae* and *Autographivirinae* subfamilies, respectively, using progressiveMauve (Fig. 4). Within the T-even phages, despite being classified as a different genus, a remarkable synteny in three large conserved regions (>10 Kb) is observed. Interestingly, Mauve alignment also demonstrated that the second block of MP1, which is similar to Pm5461, is inverted when compared to the remaining phages, suggesting a local adaptive evolution due to genetic drift. As for *Autographivirinae* phages, a more extensive re-arrangement between the sequence-blocks was observed. Overall, MP2 genome was only collinear with an MmP1 and T7. These results were also further supported using sequence dot plots (Figure S2). In the case of MP1, an interrupted but straight diagonal lines against other Teven phages, being the phage Pm5461 the one with highest score. MP2 only presented homology to phages T7 and MmP1, being the latter with an evident higher score.

While the taxonomic position of phages exclusively based on nucleotide similarity can give divergent interpretations due to the different algorithms available, phage relatedness should also be assessed with other factors such as the whole proteome and specific gene cluster organisations. Both *Morganella* phages have similar virion morphologies, genome size and number of CDSs when compared to the T4 (≈160 kb, 256–279 CDSs) and T7 archetypes (≈40 kb, 48–60 CDSs), respectively^56^. At the proteomic level, the complete set of MP1 proteins has a higher identity percentage with Pm5461 (67%), followed by T4, S16 and RB14 with proximally 50% homology. The remaining viruses fall below 50%. MP2 was compared with MmP1, presenting a high percentage of sequence identity (86%), followed by T7 (66%) and others (<54%). Taking the genomic and proteomic comparisons together, we conclude that MP1 and MP2 belong to the *Tevenvirinae* and *Autographivirinae* subfamilies, but with obvious distant relatedness to other subfamily members.

### Phylogenetic relationships

**Figure 5:**
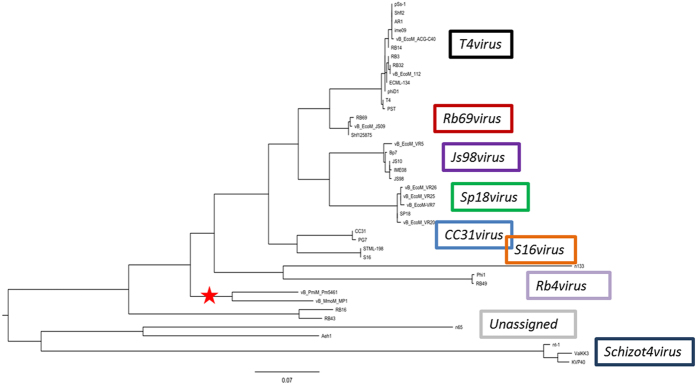
Phylogenetic analysis of MP1. Phylogenetic tree from MP1 large subunit terminase and its most closely related proteins from other closely related and distant phages. The tree was generated using the “one click” mode at phylogeny.fr.

**Figure 6:**
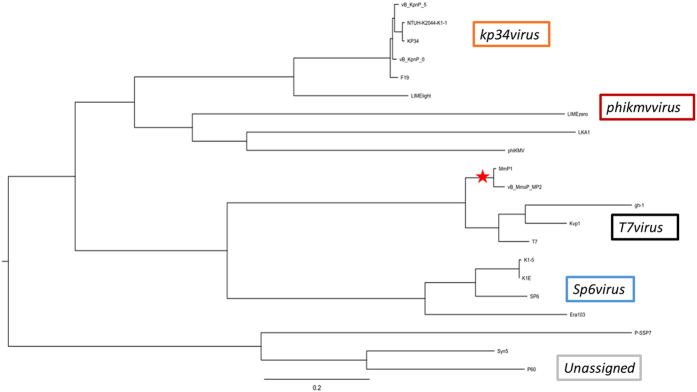
Phylogenetic analysis of MP2. Phylogenetic tree from MP2 DNA polymerase and its most closely related proteins from other closely related and distant phages. The tree was generated using the “one click” mode at phylogeny.fr.

To assess the exact taxonomic position of these two phages, the large subunit terminase proteins of MP1 and related phages, and the DNA polymerases of MP2 and related phages were analysed in “one click” mode at phylogeny.fr^46^. These genes have been previously used as marker in several phylogenetic studies of the T4- and T7-like phages^58^,^59^,^60^,^61^. The phylogenetic tree was constructed with all phages currently annotated as *Tevenvirinae* and *Autographivirinae* subfamilies along with MP1 and MP2. The resulting trees had a very good correlation with current taxonomy, meaning, every individual branch corresponded quite well with current ICTV recognized genus (Figs 5 and 6). In *Tevenvirinae*, which now has a total of eight genera, a novel branch containing the *Morganella* phage MP1 and *Proteus* phage Pm5461 was visualized. It is quite clear that MP1 and Pm5461 are phylogenetically related to each other, but significantly diverged from other tailed phages in evolution (Fig. 5). As for the *Autographivirinae* subfamily, the four genera (*T7virus, Sp6virus, Phikmvvirus* and *Kp34virus*) were also positioned on completely different branches in the resulting tree, as expected (Fig. 6). Phages MP2 and MmP1 form a distinct clade linked with the *T7virus* which is associated with a lower overall nucleotide (<10% BLASTN) and proteomic (<50% CoreGenes) identity. This suggests that both MP1 and MmP1 have diverged from a common T7-like ancestor due to genetic drift.

### Similarities and distinctions features of MP1 and MP2 genomes

**Figure 7:**
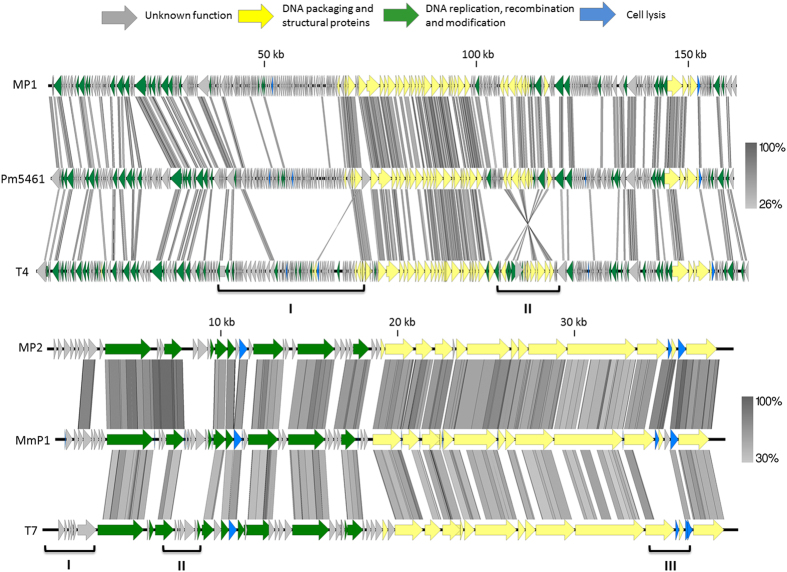
Divergence of the core gene organization of *Morganella* phages genomes. Pairwise comparisons of (**A**) MP1 and (**B**) MP2 genomes using tbBLASTX within EasyFig against the closest homolog (Pm5461 or MmP1) and the phage archetypes (T4 or T7), respectively. Arrows indicating CDSs are drawn to scale and colored in the reference genome (MP1 or MP2) according to their predicted function. Gene similarity profiles between phages are indicated in grayscale (and percentage). Major diversions are marked in roman numbers.

To individualize the similarities and differences of MP1 and MP2, we constructed comparative genome maps to show the detail diversion of the core gene organisation against phages with closer homology (Fig. 7). The starting gene and orientation of MP1 and MP2 followed the T4 and T7 convention, respectively.

MP1 alignment demonstrates short conserved regions frequently interspersed by stretches with no homology (Fig. 7A). Notably, only 85 out of 271 CDSs share >50% amino acid sequence identity with Pm5461. The homology is even lower comparatively to the T4 proteome. The middle halves of the genome, which are closely related, include most of the structural and packaging proteins (e.g. baseplate wedge subunits, tail fibers, neck wiskers, tail sheath and major capsid), which have been identified by mass spectrometry. Interestingly, halves upstream (cluster I) and downstream (cluster II) of this region are the ones that display the most significant differences. Cluster I exclusively encode leftward-oriented hypothetical genes, with the exception of two endonucleases (*gp96-97*) and the endolysin (*gp101*). Some of these proteins with unknown functions might be related to MP1 adaptation to different host ranges as previously observed with other T4-like phages^49^,^62^. Interestingly, cluster II with >10 kb long (see also green box of Fig. 7A) coding for a second group of structural genes, mostly for distal host recognition devices (baseplate tail cap, wedge and hubs) and outer capsid related proteins, is shared with Pm5461 proteome but is completely inverted on T4 and all other T-even phages. Although it is known that chromosomal inversions can alter gene expression patterns and also result in major phenotypic alterations^23^,^50^, the specific biological meaning is unknown. Nevertheless, this example illustrates well that T-even evolution is not exclusively driven by exchanging, deleting and adding of genomic segments, but also by chromosomal inversions that may play an important role as well. This seems to be particularly true in MP1, fostering the ecological divergence in *Tevenvirinae* subfamily.

In MP2, the major differences are located in the early genes cluster (cluster I), beginning of the DNA replication and recombination module (cluster II) and in the lysis cassette (cluster III) (Fig. 7B) that helped to provide phylogenetic distance between MP2 and T7. The high proteomic homology between MP2 to MmP1 is evident, but drastically decreases when compared to T7. From the early genes found (*gp1* to *gp9*), only two proteins (*gp1* and *gp3*) had homology with phages, namely *Citrobacter* phage CR8 and MmP1, with relatively low homology (<64% amino acid identity). In the remaining CDSs, no BLASP hits were found. This genomic region has been implicated with mechanisms that block the host macromolecular synthesis in T7-like phages, which in MP2 appears to be novel^60^. The RNAP, which is located in the DNA replication and recombination module, is a distinctive feature of the T7-like phages^63^,^64^, although located three CDSs ahead of T7. The MP2 RNAP has a relatively high homology to MmP1 gene (92% amino acid identity), dropping below 72% against the remaining phage proteins in the database. Three hypothetical CDSs located downwards of the DNA ligase (*gp13*) are novel. In the DNA packaging and structural protein cluster, a relatively strong homology was found, particularly against MmP1 (>70% coverage). However, mass spectrometry also allowed the identification of three unique proteins, which are probably also part of the mature virion (*gp33*-*gp34, gp39*). Notably, it seems that both MP2 and MmP1 have acquired complete novel genes coding for holin, DNA packaging and Rz-like genes (*gp47-49*), as lack of relatedness is observed to T7. This evolutionary adaptation makes sense, as while the similar endolysin found acts against a conserved peptidoglycan molecule (A1ϒ chemotype) shared by Gram-negative bacteria^65^, the acquisition of novel holins and spanins are necessary to deal with a diversified bacterial membrane structures, in this case of *M. morganii* cells. Overall, the genetic gains, losses, or replacements of MP1 and MP2 provide novel insights into the particular steps of genome divergence.

## Conclusion

We have isolated and characterized two novel representatives of phages (MP1 and MP2) that infect drug-resistant *M. morganii* clinical isolates and feature unique characteristics. The obligatorily lytic nature of MP1 and MP2, their wide host range within *M. morganii* clinical isolates and the lack of toxicity of their virions for mammalian cells makes both these phages suitable for further studies in the development of phage cocktail against drug-resistant *M. morganii* strains. Additionally, based on limited DNA sequence identity (39% as shown using BLASTN) between *Morganella* phage vB_MmoM_MP1 and *Proteus* phage vB_PmiM_Pm5461, we cannot yet recommend that ICTV create a new genus within the *Tevenvirinae*. On the other hand, the similarity between *Morganella* phages vB_MmoP_MP2 and MmP1 (70% at DNA and 86% at protein levels) strongly indicate that these two phages should be considered as species within a new genus in the *Autographivirinae*. We propose that this be called “Mmp1virus” after the first characterized isolate.

## Additional Information

**How to cite this article**: Oliveira, H. *et al*. Characterization and genomic analyses of two newly isolated *Morganella* phages define distant members among *Tevenvirinae* and *Autographivirinae* subfamilies. *Sci. Rep.* **7**, 46157; doi: 10.1038/srep46157 (2017).

**Publisher's note:** Springer Nature remains neutral with regard to jurisdictional claims in published maps and institutional affiliations.

## Supplementary Material

Supplementary Information

## Figures and Tables

**Table 1:** Bacteriophage MP1 structural proteins identified by ESI-MS/MS.

Protein	Identified function	Band N° (most abundant)	Protein MW (Da) MS/MS^a^	Protein MW (Da)^b^	N° of unique peptides	Sequence coverage, %	HHPred significant match	E-value^c^	Identitiy, %^d^
gp007	—	10	15,738	14,750	1	7.4	—	—	—
gp012	Exonuclease A	7	28,702	26,030	2	9.9	exonuclease A [*Citrobacter* phage Merlin] dCTP pyrophosphatase [*Proteus* phage vB_PmiM_Pm5461]	1E-108	66%
gp016	dCTP pyrophosphatase	9	20,529	20,190	3	18.1		5E-91	72%
gp018	—	7	33,158	32,490	5	22.9	—	—	—
gp030	RecA-like recombination	5–7 (6)	44,549	42,850	11	30.5	RecA-like recombination protein [*Serratia* phage PS2]	0.0	81%
gp038	DNA polymerase accessory protein clamp loader subunit	6–7 (6)	36,767	36,470	3	10.8	DNA polymerase accessory protein clamp loader subunit [*Proteus* phage vB_PmiM_Pm5461]	1E-155	66%
gp039	Sliding clamp	7	26,260	25,380	5	21.3	sliding clamp [*Proteus* phage vB_PmiM_Pm5461]	4E-78	55%
gp042	Hypothetical protein	12	9,690	9,580	4	40.0	hypothetical protein [*Morganella morganii*]	2E-22	53%
gp049	Hypothetical protein	11	14,126	13,190	1	15.8	hypothetical protein Pm5461_055 [*Proteus* phage vB_PmiM_Pm5461]	7E-17	44%
gp051	Sigma factor for late transcription	9	21,122	20,500	4	26.4	sigma factor for late transcription [*Proteus* phage vB_PmiM_Pm5461]	7E-82	70%
gp059	Hypothetical protein	11	15,176	14,750	6	46.3	hypothetical protein Aeh1p318 [*Aeromonas* phage Aeh1]	3E-11	55%
gp069	Thioredoxin	7	40,600	39,370	2	6.6	thioredoxin [*Salmonella* phage STML-198]	0.0	80%
gp071	Hypothetical protein	10	19,622	17,150	4	29.8	hypothetical protein Pm5461_072 [*Proteus* phage vB_PmiM_Pm5461]	2E-24	39%
gp073	—	10–11 (10)	20,635	19,200	8	44.3	—	—	—
gp100	Hypothetical protein	11	17,714	16,480	3	17.0	hypothetical protein Pm5461_100 [*Proteus* phage vB_PmiM_Pm5461]	5E-42	60%
gp101	Endolysin	11	14,738	14,480	3	33.8	peptidase M15 [Escherichia coli]	3E-54	71%
gp113	Hypothetical protein	7-11 (8)	23,179	22,310	6	35.3	hypothetical protein Pm5461_106 [*Proteus* phage vB_PmiM_Pm5461]	2E-42	43%
gp117	Hypothetical protein	10	16,842	16,160	4	28.9	hypothetical protein VR7_gp147 [Enterobacteria phage vB_EcoM-VR7]	9E-26	45%
gp119	Hypothetical protein	10	17,175	16,260	2	14.6	hypothetical protein SP18_gp147 [*Shigella* phage SP18]	5E-40	52%
gp136	Hypothetical protein	11	11,613	11,210	6	59.0	hypothetical protein STP4a_129 [*Salmonella* phage STP4-a]	2E-17	47%
gp137	Hypothetical protein	10	16,966	16,880	2	16.7	gp57B conserved hypothetical protein [Enterobacteria phage T4]	1E-79	77%
gp139	dNMP kinase	7	28,387	26,640	5	18.9	dNMP kinase [*Proteus* phage vB_PmiM_Pm5461]	1E-56	47%
gp140	Tail completion and sheath stabilizer	9	22,238	21,840	2	11.1	gp3 tail completion and sheath stabilizer protein [Enterobacteria phage CC31]	5E-80	58%
gp141	DNA end protector	7	31,464	30,950	3	9.2	DNA end protector protein [*Proteus* phage vB_PmiM_Pm5461]	4E-139	73%
gp147	Baseplate wedge subunit	2–3	72,921	72,370	18	37.9	baseplate wedge subunit [*Proteus* phage vB_PmiM_Pm5461]	0.0	72%
gp148	Baseplate wedge subunit	1	122,856	121,220	1	0,9	baseplate wedge subunit [*Proteus* phage vB_PmiM_Pm5461]	0.0	69%
gp149	Baseplate wedge tail fiber connector	5	39,290	38,630	2	7.4	baseplate wedge tail fiber connector [*Proteus* phage vB_PmiM_Pm5461]	0.0	77%
gp150	Baseplate wedge subunit and tail pin	7	31,824	31,340	2	6.3	baseplate wedge subunit and tail pin [*Proteus* phage vB_PmiM_Pm5461]	5E-135	67%
gp151	Baseplate wedge subunit and tail pin	2–3	67,569	67,110	14	33.9	baseplate wedge subunit and tail pin [*Citrobacter* phage Moon]	0.0	48%
gp152	Baseplate wedge subunit and tail pin	8	26,233	23,260	2	8,9	baseplate wedge subunit and tail pin [*Proteus* phage vB_PmiM_Pm5461]	3E-45	45%
gp153	Short tail fibers	5	48,108	47,610	9	27.8	short tail fibers protein [*Proteus* phage vB_PmiM_Pm5461]	3E-133	49%
gp158	Tail sheath stabilization	6	32,675	32,380	1	4.0	tail sheath stabilization protein [*Proteus* phage vB_PmiM_Pm5461]	8E-134	68%
gp161	Tail sheath	1–3,7 (3)	73,206	72,620	26	51.3	tail sheath protein [*Proteus* phage vB_PmiM_Pm5461]	0.0	76%
gp162	Tail tube	9	20,855	18,570	4	21.3	tail tube protein [*Proteus* phage vB_PmiM_Pm5461]	3E-97	79%
gp163	Portal vertex of head	2–4 (3)	61,044	60,300	16	41.0	portal vertex of head [*Proteus* phage vB_PmiM_Pm5461]	0.0	68%
gp165	Prohead core	11	17,074	16,190	1	6.9	prohead core protein [*Proteus* phage vB_PmiM_Pm5461]	3E-51	70%
gp166	Prohead core scaffold and Protease	10	24,248	23,130	3	16.1	prohead core scaffold protein and protease [*Proteus* phage vB_PmiM_Pm5461]	2E-111	79%
gp167	Prohead core scaffold	2–11 (6)	32,335	30,360	17	77.9	prohead core scaffold protein [*Proteus* phage vB_PmiM_Pm5461]	3E-77	56%
gp168	Major capsid	1–11 (4)	55,998	56,010	23	60.24	major capsid protein [*Proteus* phage vB_PmiM_Pm5461]	0.0	81%
gp169	Head vertex	4–6 (5)	46,883	46,160	21	66.5	head vertex protein [*Proteus* phage vB_PmiM_Pm5461]	0.0	79%
gp187	Baseplate tail tube initiator	7	33,526	33,420	4	16.1	baseplate tail tube initiator [*Proteus* phage vB_PmiM_Pm5461]	7E-174	79%
gp189	Baseplate hub subunit and tail length determinator	2–3	63,213	62,730	2	4.4	baseplate hub subunit and tail length determinator [*Proteus* phage vB_PmiM_Pm5461]	3E-119	41%
gp190	Baseplate hub distal subunit	9	19,816	18,180	3	18.5	baseplate hub distal subunit [*Proteus* phage vB_PmiM_Pm5461]	3E-62	59%
gp191	Baseplate hub subunit	5	45,005	44,550	2	7.4	baseplate hub subunit [*Proteus* phage vB_PmiM_Pm5461]	0.0	68%
gp194	Baseplate wedge subunit	11	14,497	14,380	2	14.0	baseplate wedge subunit [*Proteus* phage vB_PmiM_Pm5461]	9E-56	66%
gp195	recombination, repair and ssDNA binding protein	10–11	17,680	15,740	3	22.4	recombination, repair and ssDNA binding protein [*Proteus* phage vB_PmiM_Pm5461]	3E-65	71%
gp201	Head outer capsid	5–6 (6)	40,233	39,710	13	41.3	head outer capsid protein [*Proteus* phage vB_PmiM_Pm5461]	1E-176	68%
gp205	RNA polymerase ADP-ribosylase	1–3,5,7 (3)	79,188	77,210	25	35.3	RNA polymerase ADP-ribosylase [*Proteus* phage vB_PmiM_Pm5461]	0.0	49%
gp206	DNA ligase	4,7 (7)	55,737	55,740	10	27.4	DNA ligase [*Proteus* phage vB_PmiM_Pm5461]	0.0	61%
gp207	Hypothetical protein	8	24,524	24,410	4	21.6	hypothetical protein Pm5461_192 [*Proteus* phage vB_PmiM_Pm5461]	3E-126	84%
gp231	RNA ligase 1 and tail fiber attachment catalyst	5–7,10 (5)	45,404	42,480	8	25.2	RNA ligase 1 and tail fiber attachment catalyst [*Proteus* phage vB_PmiM_Pm5461]	5E-147	58%
gp244	Hypothetical protein	10	16,419	16,420	2	15.7	hypothetical protein Pm5461_225 [*Proteus* phage vB_PmiM_Pm5461]	3E-11	31%
gp249	dsDNA binding protein	11	11,382	10,360	5	41.8	dsDNA binding protein [*Proteus* phage vB_PmiM_Pm5461]	8E-41	75%
gp251	Long tail fiber proximal subunit	1–3 (3)	134,949	134,620	12	14.0	long tail fiber proximal subunit [*Proteus* phage vB_PmiM_Pm5461]	0.0	60%
gp253	Long tail fiber distal connector	10	19,223	17,300	3	30.7	long tail fiber distal connector [*Proteus* phage vB_PmiM_Pm5461]	2E-24	40%
gp254	Long tail fiber distal subunit	4	61,920	61,700	6	13.9	long tail fiber distal subunit [*Proteus* phage vB_PmiM_Pm5461]	2E-22	46%
gp266	DNA topoisomerase	4–5 (5)	54,442	51,720	3	6.7	DNA topoisomerase [*Proteus* phage vB_PmiM_Pm5461]	0.0	63%
gp267	Nucleoid disruption protein	10	17,925	17,410	1	7.1	nucleoid disruption protein [*Proteus* phage vB_PmiM_Pm5461]	9E-25	44%

^a^Molecular weight (MW) measured via mass spectrometry.

^b^Molecular weight (MW) predicted on the basis of amino acid sequence.

^c^HHpred E-value of the most significant matching protein model.

^d^HHpred percent sequence identity for a pairwise aa alignment with the template protein.

Identified phage proteins are listed below. Their mass and SDS-PAGE band have been indicated, as the number of identified peptides and the complete protein coverage %. It should be noted that Identifications with a low abundance of peptides (<4) could be the result of protein impurities remaining in the samples.

**Table 2:** Bacteriophage MP2 structural proteins identified by ESI-MS/MS.

Protein	Identified function	Band N° (most abundant)	Protein MW (Da) MS/MS^a^	Protein MW (Da)^b^	N° of unique peptides	Sequence coverage, %	HHPred significant match	E-value^c^	Identitiy, %^d^
gp06	—	12	12,086	11,550	2	25.2	—	—	—
gp15	Hypothetical protein	7	22,670	21,720	1	4.5	hypothetical protein MmP1_gp16 [*Morganella* phage MmP1]	3E-48	53
gp18	Single-stranded DNA-binding protein	6–8 (6)	26,990	26,470	10	63.0	single-stranded DNA-binding protein [*Morganella* phage MmP1]	2E-133	87
gp21	Endolysin	10	17,690	17,370	2	12.7	lysozyme [*Morganella* phage MmP1]	8E-102	93
gp25	Hypothetical protein	11	8,294	7,830	1	21.6	hypothetical protein MmP1_gp24 [*Morganella* phage MmP1]	3E-24	93
gp29	—	11–12 (11)	13,080	10,520	4	38.5	—	—	—
gp32	Exonuclease	5–6	36,568	35,060	5	19.4	exonuclease [*Morganella* phage MmP1]	0.0	90
gp33	Hypothetical protein	12	9,793	9,360	1	15.1	hypothetical protein MmP1_gp32 [*Morganella* phage MmP1]	3E-17	74
gp34	Hypothetical protein	10	8,919	8,440	4	59.0	hypothetical protein MmP1_gp33 [*Morganella* phage MmP1]	3E-23	70
gp35	Host Range Protein	10	8,427	7,930	3	60.5	hypothetical protein [*Escherichia* phage CICC 80001]	2E-4	46
gp36	Head-to-tail joining protein	1–5 (3)	59,543	59,350	32	75.4	head-to-tail joining protein [*Morganella* phage MmP1]	0.0	92
gp37	Capsid assembly protein	1–7 (5)	38,204	35,430	15	56.9	capsid assembly protein [*Morganella* phage MmP1]	0.0	80
gp38	Major capsid protein	1–12 (5)	36,358	36,230	22	76.5	major capsid protein [*Morganella* phage MmP1]; minor capsid protein [*Morganella* phage MmP1]	0.0	89
gp39	Hypothetical protein	1,4–7 (5)	7,693	4,690	1	31.4	hypothetical protein MmP1_gp37 [*Morganella* phage MmP1]; minor capsid protein [*Morganella* phage MmP1]	7E-5	80
gp40	Tail tubular protein A	7–8 (7)	23,027	22,090	7	59.4	tail tubular protein A [*Morganella* phage MmP1]	6E-122	86
gp41	Tail tubular protein B	1–5,7,10 (2)	90,762	90,770	32	47.9	tail tubular protein B [*Morganella* phage MmP1]	0.0	88
gp43	Internal virio + n protein B	8	21,669	20,410	10	50.5	internal virion protein B [*Morganella* phage MmP1]	1E-87	84
gp44	Internal virion protein C	1–4,6–7,10 (3)	82,793	82,570	38	54.4	internal virion protein C [*Morganella* phage MmP1]	0.0	76
gp45	Internal virion protein D	1–7 (2)	144,296	143,870	58	59.2	internal virion protein D [*Morganella* phage MmP1]	0.0	70
gp46	Tail fiber protein	1–7,10,12 (3)	63,591	63,260	31	64.5	tail fiber protein [*Morganella* phage MmP1]	0.0	72
gp50	DNA packaging protein B	1,3 (3)	68,739	66,550	7	11.9	DNA maturase B [*Morganella* phage MmP1]	0.0	92

^a^Molecular weight (MW) measured via mass spectrometry.

^b^Molecular weight (MW) predicted on the basis of amino acid sequence.

^c^HHpred E-value of the most significant matching protein model.

^d^HHpred percent sequence identity for a pairwise aa alignment with the template protein.

Identified phage proteins are listed below. Their mass and SDS-PAGE band have been indicated, as the number of identified peptides and the complete protein coverage %. It should be noted that Identifications with a low abundance of peptides (<4) could be the result of protein impurities remaining in the samples.
