# Supplementary material for: Characterization and genomic analyses of two newly isolated Morganella phages define distant members among Tevenvirinae and Autographivirinae subfamilies
Source: Sci Rep. 2017 Apr 7;7:46157. doi: 10.1038/srep46157 (PMC5384007; doi:10.1038/srep46157)

Characterization and genomic analyses of two newly isolated *Morganella* phages define distant members among *Tevenvirinae* and *Autographivirinae* subfamilies

Hugo Oliveira[1]†, Graça Pinto[1]†, Ana Oliveira[1], Jean-Paul Noben[2], Hanne Hendrix[3], Rob Lavigne[3], Małgorzata Łobocka[4,5] Andrew M. Kropinski[6] and Joana Azeredo[1*]

†Equal contribution

Table S1. ***Morganella morganii*** **phages MP1 and MP2 lytic spectra and efficiency of plating.**

Species	Strain	Origin	Patient gender	Antibiogram	MP1 Infectivity	MP1 EOP*	MP2 Infectivity	MP2 EOP*
Morganella morganii	M1	CDC 4195-69 (from Salmonella Genetic Stock centre)			+	Low	-	
	M2	Urine	Female	AM, AMC, CXM, SXT, TZP, CT, TZ, GM, NN, AN, AmpC	-		+	High
	M3	Urine	Female	AM, AMC, CXM, CIP, LVX, FM	+	High	-	
	M4	Urine	Female	FM, AM, AMC, CXM, CIP, LVX, SXT	+	High	-	
	M5	Urine	Male	AM, AMC, CXM, SXT, TZP, CT, TZ, CIP, GM, NN, AN, AmpC	-		+	High
	M6	Faeces	Female	Unknown	+	LFW	+	Low
	M7	Urine	Female	AM, AMC, CXM, SXT, TZP, CT, TZ, CIP, GM, NN, AN, AmpC	+	High	+	High
	M8	Urine	Female	AM, AMC, CXM, FM	-		+	High
	M9	Urine	Male	AM, AMC, CXM, FM	+	High	-	
	M10	Faeces	Female	Unknown	+	Low	-	
	M11	Urine	Female	AM, AMC, CXM, GM, FM, NN, SXT	-		+	High
	M12	Urine	Female	AM, AMC, CXM, SXT, TZP, CT, TZ, GM, NN, AN, AmpC	-		+	High
	M13	Urine	Male	AM, AMC, FM, AmpC	+	LFW	+	High
	M14	Cerebrospinal	Male	AM, AMC, CXM, SXT, TZP, CT, TZ, CIP, GM, NN, AN, AmpC	-		-	
	M15	Urine	Male	AM, AMC, CXM, CT, TZ, TZP	-		-	
	M16	Urine	Male	AM, AMC, CXM	+	LFW	+	High
	M17	Urine	Male	AM, AMC, CXM, FM, SXT, CIP, TZ, CT, AmpC	+	LFW	+	Low
	M18	Pus	Male	AM, AMC, CXM, SXT, TZP, CT, TZ, AmpC	-		-	
	M19	Urine	Male	AM, AMC, CXM, SXT, TZP, AmpC	+	LFW	-	
	M20	sputum	Male	AM, AMC, CXM, SXT, FM, CIP, LVX	+	High	-	
	M21	Urine	Female	AM, AMC, CXM, FM	+	Low	+	LFW
	M22	Urine	Male	AM, AMC	+	LFW	-	
	M23	Urine	Male	AM, AMC, CXM, CIP, LVX, SXT	+	LFW	+	High
	M24	Expectoration	Female	AM, AMC, CXM	+	LFW	+	Low
	M25	Urine	Female	AM, AMC, FM, CIP	+	Low	+	High
	M26	Urine	Female	AM, AMC, CXM, SXT, TZP, CT, TZ, CIP, GM, NN	+	LFW	-	
	M27	Urine	Male	AM, AMC, CIP, LVX, FM	+	High	+	High
Citrobacter freundii	CF	EC592 (from Salmonella Genetic Stock centre)			-		-	
Citrobacter koseri	CK	Pus	Male	AM	-		-	
Providencia stuartii	PS	Urine	Female	AM, AMC, CXM	-		-	
Proteus mirabilis	PM	CECT 4101 (from American Type Culture centre)			-		-	
Proteus vulgaris	PV	CECT 174 (from American Type Culture centre)			-		-	
Escherichia coli	EC	CECT 432 (from American Type Culture centre)			-		-	

[1] Collection strains: M1 - SGSC 5703; CF - SGSC 5345; PM - CECT 4168; PV - CECT 174; EC - CECT 432

[2] Antibiotic resistances: M - ampicillin; AMC - amoxicillin/clavulanic acid; CXM - cefuroxime; SXT - cotrimoxazole; TZP - piperacillin/tazobactam; MEM - meropenem; CT - cefotaxime; TZ - ceftazidime; CIP - ciprofloxacin; GM - gentamicin; NN - tobramycin; AN - amikacin; ESBL - Extended-Spectrum Beta-lactamase producing bacteria; AmpC - beta-lactamases producing bacteria

[3] The EOP was recorded as high and low representing higher and lower than 1 % respectively and marked as LFW for lysis from without events

Table S2. Features of MP1 predicted CDSs. For each CDS the transcription start and stop position, and the coding strand is given. At protein level the corresponding gene product size molecular weight and pI as well as the homolog predicted function, homology values and motifs are shown.

			Predicted protein						
ORF (+/-)	**Start (bp)**	**Stop (bp)**	**Size (aa)**	**MW (Kda)**	**pI**	**Best species hit**	**Putative function (accession no.)**	**E-value (% identify)**	**Motifs**
1/-	15	2177	720	8303	816	Proteus phage vB_PmiM_Pm5461	rIIA protein	0.0 (45)	-
2/-	2131	2370	79	9.26	5.66	-	-	-	-
3/-	2373	2561	62	7.05	4.60	-	-	-	-
4/-	2558	4375	605	68.46	6.71	Acinetobacter phage Acj61	DNA topoisomerase subunit (YP_004009629.1)	0.0 (67)	HATPase_c (PF02518.23); DNA_gyraseB (PF00204.22)
5/-	4356	4700	114	13.81	6.52	Proteus phage vB_PmiM	Hypothetical protein (AKA61870.1)	600E-16 (41)	-
6/-	4736	5137	133	15.34	5.00	-	-	-	-
7/-	5134	5514	126	14.75	4.40	-	-	-	-
8/-	5511	6197	228	26.47	4.56	-	-	-	-
9/-	6194	6817	207	23.68	4.19	Proteus phage vB_PmiM	Hypothetical protein (AKA61874.1)	100E-37 (40)	-
10/-	6825	7013	62	7.33	4.27	-	-	-	-
11/-	7076	7777	233	26.78	4.24	-	-	-	-
12/-	7779	8468	229	26.03	5.01	Citrobacter phage Merlin	Exonuclease A (AKU43661.1)	100E-108 (66)	DUF5051 (PF16473.2)
13/-	8471	9796	441	50.66	6.73	Proteus phage vB_PmiM	DNA helicase (AKA61877.1)	0.0 (59)	AAA_30 (PF13604.3); UvrD_C_2 (PF13538.3)
14/-	9793	10074	93	11.2	10.02	Acinetobacter phage Ac42	Hypothetical protein (YP_004009387.1)	700E-10 (34)	-
15/-	10049	10633	194	23.02	10.16	Proteus phage vB_PmiM	Anti-sigma factor (AKA61879.1)	600E-24 (34)	-
16/-	10637	11161	174	20.19	4.78	Proteus phage vB_PmiM	dCTP pyrophosphatase (AKA61881.1)	500E-91 (72)	-
17/-	11220	11768	182	21.06	5.86	Proteus phage vB_PmiM	Hypothetical protein (AKA61883.1)	100E-07 (39)	-
18/-	11770	12591	273	32.49	7.12	-	-	-	-
19/-	12591	13034	147	17.14	5.00	-	-	-	-
20/-	13037	13447	136	15.8	4.96	Proteus phage vB_PmiM	Hypothetical protein (AKA61884.1)	100E-46 (56)	-
21/-	13458	14246	262	30.33	6.00	Proteus phage vB_PmiM	Hypothetical protein (AKA61885.1)	500E-132 (69)	-
22/-	14308	14619	103	12.15	4.65	-	-	-	-
23/-	14710	14877	55	6.53	3.82	-	-	-	-

24/-	14867	15532	221	25.62	4.69	-	-	-	-
25/-	15536	15937	133	15.62	5.25	-	-	-	-
26/-	15941	16966	341	40.02	8.99	Proteus phage vB_PmiM	DNA primase subunit (AKA61888.1)	200E-153 (63)	-
27/-	17098	17544	148	16.43	9.99	Shigella phage Shf125875	Hypothetical protein (YP_009100573.1)	400E-60 (65)	Gp23 (PF07068.8)
28/-	17541	18935	464	52.28	5.01	Proteus phage vB_PmiM	DNA helicase (AKA61890.1)	0.0 (73)	DnaB_C (PF03796.12)
29/-	18929	19267	112	13.04	4.10	Proteus phage vB_PmiM	Head vertex assembly chaperone (AKA61891.1)	200E-27 (52)	Phage_head_chap (PF11113.5)
30/-	19301	20446	381	42.85	5.54	Serratia phage PS2	RecA-like recombination (YP_009030086.1)	0.0 (81)	RecA (PF00154.18)
31/-	20500	21030	176	20.83	4.86	Yersinia phage phiR1-RT	Hypothetical protein (YP_007235878.1)	200E-52 (54)	-
32/-	21027	21239	70	7.98	10.06	-	-	-	1 TMD
33/-	21236	21667	143	16.72	9.64	Proteus phage vB_PmiM	Hypothetical protein (AKA61905.1)	200E-15 (37)	1 SP
34/-	21719	24436	905	104.57	6.14	Proteus phage vB_PmiM	DNA polymerase (AKA61906.1)	0.0 (73)	DNA_pol_B_exo1(PF03104.16); DNA_pol_B (PF00136.18)
35/-	24492	24851	119	14.01	8.71	Proteus phage vB_PmiM	Translational repressor (AKA61908.1)	700E-60 (76)	Translat_reg (PF01818.14)
36/-	24876	25040	54	6.13	9.58	-	-	-	-
37/-	25043	25624	193	23.07	5.38	Proteus phage vB_PmiM	DNA polymerase accessory protein clamp loader subunit (AKA61909.1)	300E-69 (56)	Phage_clamp_A (PF16790.2)
38/-	25617	26585	322	36.47	7.27	Proteus phage vB_PmiM	DNA polymerase accessory protein clamp loader subunit (AKA61910.1)	100E-155 (66)	AAA (PF00004.26)
39/-	26595	27281	228	25.38	4.52	Proteus phage vB_PmiM	Sliding clamp (AKA61911.1)	400E-78 (55)	DNA_PPF (PF02916.12); gp45-slide_C (PF09116.7)
40/-	27286	27606	106	12.51	5.77	Enterobacteria phage RB49	RNA polymerase biding (NP_891603.1)	400E-12 (43)	Phage_RpbA (PF10789.6)
41/-	27599	27787	62	7.4	4.15	Serratia phage PS2	Hypothetical protein (YP_009030106.1)	200E-10 (50)	-
42/-	27774	28028	84	9.58	4.79	Morganella morganii	Hypothetical protein (WP_046394122.1)	200E-22 (53)	-
43/-	28018	29709	563	64.1	7.94	Proteus phage vB_PmiM	Recombination endonuclease subunit (AKA61914.1)	0.0 (60)	AAA_23 (PF13476.3)
44/-	29706	30707	333	38.59	5.16	Proteus phage vB_PmiM	Recombination endonuclease subunit (AKA61915.1)	800E-169 (66)	-
45/-	30725	31033	102	11.9	4.45	-	-	-	-
46/-	31042	31353	103	11.97	8.21	-	-	-	-
47/-	31350	31568	72	8.71	9.71	-	-	-	-
48/-	31568	31723	51	6.08	6.60	-	-	-	-
49/-	31728	32066	112	13.19	5.28	Proteus phage vB_PmiM	Hypothetical protein (AKA61917.1)	700E-17 (44)	DUF2654 (PF10849.5)
50/-	32044	32250	68	8.12	4.57	-	-	-	-
51/-	32243	32761	172	20.5	4.53	Proteus phage vB_PmiM	Sigma factor for late transcription (AKA61918.1)	700E-82 (70)	-
52/-	32929	34356	475	54.81	8.80	Proteus phage vB_PmiM	Hypothetical protein (AKA61919.1)	0.0 (69)	-

53/-	34366	34659	97	11.32	6.89	Enterobacteria phage Bp7	Hypothetical protein (YP_007004107.1)	700E-20 (46)	-
54/-	34652	34927	91	10.59	5.14	Proteus phage vB_PmiM	Glutaredoxin (AKA61921.1)	200E-37 (69)	-
55/-	34924	35196	90	10.15	3.72	-	-	-	-
56/-	35196	35447	83	9.71	3.90	-	-	-	-
57/-	35444	35695	83	10.06	4.52	-	-	-	-
58/-	35692	36177	161	18.74	7.45	Enterobacteria phage T4	NrdG anaerobic NTP reductase small subunit (NP_049688.1)	300E-56 (56)	Fer4_12 (PF13353.3)
59/-	36216	36611	131	14.75	4.64	Aeromonas phage Aeh1	Hypothetical protein (NP_944196.1)	300E-11 (55)	-
60/-	36608	39385	925	104.83	7.03	Vibrio phage PVo5	Anaerobic NTP reductase large subunit (AKY02222.1)	0.0 (49)	NRDD (PF13597.3)
61/-	39382	39852	156	18.24	8.45	Proteus phage vB_PmiM	Recombinase endonuclease VII (AKA61926.1)	100E-79 (74)	Endonuclease_7 (PF02945.12); Endonuc-dimeris (PF09124.7)
62/-	39891	40079	62	7.2	4.09	-	-	-	-
63/-	40070	40336	88	9.98	6.23	Proteus phage vB_PmiM	Thioredoxin (AKA61928.1)	700E-29 (56)	Glutaredoxin (PF00462.21)
64/-	40333	40632	99	11.23	5.04	-	-	-	-
65/-	40629	40874	81	9.31	4.22	-	-	-	-
66/-	41089	41316	75	8.93	9.50	-	-	-	2 TMD; 1 SP
67/-	41313	41540	75	9.03	8.49	Enterobacteria phage T4	Hypothetical protein (NP_049700.1)	300E-18 (64)	-
68/-	41542	42126	194	22.72	7.63	Xenorhabdus griffiniae	Hypothetical protein (WP_047771056.1)	400E-17 (36)	-
69/-	42128	43144	338	39.37	6.68	Salmonella phage STML-198	Thioredoxin (YP_009148083.1)	0.0 (80)	Nuc-transf (PF10127.6)
70/-	43141	43485	114	13.5	5.72	-	-	-	1 SP
71/-	43482	43931	149	17.15	8.77	Proteus phage vB_PmiM	Hypothetical protein (AKA61934.1)	200E-24 (39)	-
72/-	43921	44142	73	8.59	4.48	Proteus phage vB_PmiM_Pm5461	Hypothetical protein (AKA61935.1)	500E-17 (55)	-
73/-	44135	44650	171	19.2	7.81	-	-	-	-
74/-	44652	44867	71	8.2	3.68	-	-	-	-
75/-	44854	44979	41	4.93	10.97	Enterobacteria phage vB_EcoM	Hypothetical protein (YP_004063951.1)	300E-09 (85)	-
76/-	44981	45718	245	27.89	9.35	Proteus phage vB_PmiM	Hypothetical protein (AKA61938.1)	100E-17 (28)	3 TMD
77/-	45729	45953	74	8.85	4.24	-	-	-	-
78/-	45955	46104	49	5.55	8.55	-	-	-	-
79/-	46094	46414	106	12.49	5.51	-	-	-	-
80/-	46414	46656	80	9.11	4.24	-	-	-	1 SP
80A/-	46653	46847	64	744	952	Citrobacter phage Merlin	Immunity to superinfection protein	400E-9 (50)	2 TMD; Imm_superinfect (PF14373.3)
81/-	46856	47089	77	8.68	4.96	-	-	-	-

82/-	47086	47589	167	19.62	4.32	-	-	-	-
83/-	47597	47758	53	6.23	9.97	Proteus phage vB_PmiM	Hypothetical protein (AKA61947.1)	200E-12 (61)	-
84/-	47755	48033	92	10.44	9.77	-	-	-	-
85/-	48135	48410	91	10.53	4.81	Proteus phage vB_PmiM	Hypothetical protein (AKA61950.1)	500E-19 (47)	-
86/-	48487	48762	91	10.23	4.33	Enterobacteria phage CC31	Lysis inhibition (YP_004009968.1)	300E-18 (44)	1 SP
87/-	48775	48978	67	7.97	10.16	Edwardsiella phage PEi20	Hypothetical protein (BAQ22762.1)	200E-04 (37)	-
88/-	49001	49579	192	21.79	6.35	Pectobacterium bacteriophage PM2	Thymidine kinase (AHY25080.1)	100E-89 (65)	TK (PF00265.15)
89/-	49581	49760	59	6.98	10.31	-	-	-	-
90/-	49784	50047	87	10.11	6.48	Enterobacteriaceae	Hypothetical protein (WP_008786603.1)	300E-10 (38)	-
91/-	50044	50313	89	10.78	9.04	Escherichia phage vB_EcoS	Hypothetical protein (YP_009031684.1)	100E-07 (39)	-
92/-	50304	50525	73	8.88	9.60	-	-	-	2 TMD
93/-	50522	50968	148	17.11	8.85	Proteus phage vB_PmiM	Hypothetical protein (AKA61957.1)	400E-63 (63)	1 SP
94/-	50965	51213	82	9.12	4.07	Proteus phage vB_PmiM	tRNA synthetase modifier (AKA61958.1)	300E-26 (56)	-
95/-	51267	51821	184	21.66	8.68	Proteus phage vB_PmiM	Hypothetical protein (AKA61959.1)	700E-62 (58)	1 SP
96/-	51818	52255	145	17.44	9.51	Proteus phage vB_PmiM	site-specific RNA endonuclease (AKA61960.1)	300E-34 (46)	REGB_T4 (PF10715.6)
97/-	52257	52652	131	15.38	10.08	Proteus phage vB_PmiM	Endonuclease V (AKA61961.1)	400E-69 (78)	Pyr_excise (PF03013.11)
98/-	52707	53108	133	14.99	6.00	Klebsiella phage JD18	Hypothetical protein (AKY01977.1)	400E-39 (58)	Gly_radical (PF01228.18)
99/-	53246	53626	126	15.1	7.66	Proteus phage vB_PmiM	Hypothetical protein (AKA61964.1)	700E-07 (33)	-
100/-	53680	54111	143	16.48	5.73	Proteus phage vB_PmiM	Hypothetical protein (AKA61965.1)	500E-42 (60)	-
101/-	54142	54525	127	14.48	9.33	Escherichia coli	peptidase M15 (WP_000002675.1)	300E-54 (71)	VanY (PF02557); Peptidase_M15_4 (PF13539.3)
102/-	54559	54966	135	15.99	4.58	Proteus phage vB_PmiM	Hypothetical protein (AKA61967.1)	300E-07 (38)	-
103/-	54968	55240	90	10.16	7.25	-	-	-	-
104/-	55230	55643	137	15.67	5.23	Proteus phage vB_PmiM	Nudix hydrolase (AKA61968.1)	600E-44 (57)	4 TMD; NUDIX (PF00293.25)
105/-	55873	56097	74	8.75	3.98	-	-	-	-
106/-	56099	56629	176	20.3	8.57	-	-	-	-
107/-	56619	57122	167	20.21	6.29	-	-	-	-
108/-	57119	57595	158	18.65	8.40	-	-	-	-
109/-	57592	58026	144	16.73	9.11	-	-	-	3 TMD
110/-	58023	58355	110	12.84	9.53	-	-	-	2 TMD

111/-	58352	58624	90	10.73	10.48	-	-	-	1 TMD; 1 SP
112/-	58624	58947	107	12.27	9.75	-	-	-	2 TMD; 1 SP
113/-	58989	59585	198	22.31	5.82	Proteus phage vB_PmiM	Hypothetical protein (AKA61971.1)	200E-42 (43)	-
114/-	59597	59794	65	7.15	9.88	Enterobacter phage EcP1	Hypothetical protein (YP_007003127.1)	100E-06 (35)	1 TMD
115/-	59864	60136	90	10.56	4.51	Proteus phage vB_PmiM	Hypothetical protein (AKA61973.1)	300E-49 (83)	-
116/-	60221	60463	80	8.95	8.50	Proteus phage vB_PmiM	Hypothetical protein (AKA61976.1)	200E-44 (84)	-
117/-	60505	60945	146	16.16	5.23	Enterobacteria phage vB_EcoM-VR7	Hypothetical protein (YP_004063828.1)	900E-26 (45)	-
118/-	61046	61315	89	10	4.62	Proteus phage vB_PmiM	Hypothetical protein (AKA61973.1)	200E-37 (69)	-
119/-	61398	61829	143	16.26	10.45	Shigella phage SP18	Hypothetical protein (YP_003934772.1)	500E-40 (52)	-
120/-	61947	62297	116	13.48	4.85	Proteus phage vB_PmiM	Hypothetical protein (AKA61978.1)	100E-38 (57)	-
121/-	62298	62489	63	7.36	4.17	-	-	-	-
122/-	62499	62828	109	12.64	5.66	Thermaerobacter marianensis DSM 12885	Spore coat assembly (ADU50651.1)	800E-07 (58)	LysM (PF01476.17)
123/-	63002	63340	112	12.99	4.07	Proteus phage vB_PmiM	Hypothetical protein (AKA61981.1)	100E-31 (55)	-
124/-	63350	63649	99	11.65	4.04	-	-	-	-
125/-	63668	63847	59	6.93	10.33	-	-	-	-
126/-	64171	64419	82	9.94	9.86	-	-	-	1 TMD
127/-	64534	65061	175	20.97	9.62	Edwardsiella phage PEi20	Hypothetical protein (BAQ22801.1)	700E-104 (84)	T5orf172 (PF10544.6)
128/-	65314	65478	54	6.45	10.69	-	-	-	-
129/-	65667	65975	102	11.75	4.15	-	-	-	-
130/-	65993	66379	128	14.14	7.44	Proteus phage vB_PmiM	Hypothetical protein (AKA61987.1)	100E-63 (70)	1 SP
131/-	66578	66742	54	6.59	10.24	-	-	-	-
132/-	66835	66948	37	4.11	10.13	-	-	-	-
133/-	67012	67452	146	17.07	4.44	Klebsiella phage JD18	Hypothetical protein (AKY01997.1)	400E-23 (38)	-
134/-	67449	67709	86	10.22	8.47	-	-	-	-
135/-	67718	67987	89	10.03	9.73	-	-	-	-
136/-	68008	68298	96	11.21	8.96	Salmonella phage STP4-a	Hypothetical protein (YP_009126336.1)	200E-17 (47)	-
137/-	68355	68804	149	16.88	6.51	Enterobacteria phage T4	Hypothetical protein (NP_049750.1)	100E-79 (77)	-
138/-	68797	69069	90	10.24	3.88	-	-	-	-
139/-	69020	69706	228	26.64	6.52	Proteus phage vB_PmiM	dNMP kinase (AKA61994.1)	100E-56 (47)	-

140/-	69709	70296	195	21.84	4.82	Enterobacteria phage CC31	Tail completion and sheath stabilizer (YP_004010008.1)	500E-80 (58)	Phage_T4_gp19 (PF06841.9)
141/-	70513	71319	268	30.95	10.61	Proteus phage vB_PmiM	DNA end protector (AKA61996.1)	400E-139 (73)	-
142/+	71321	71770	149	17.84	10.32	Serratia phage PS2	Head completion (YP_009030206.1)	500E-77 (73)	-
143/+	71829	72380	183	21.61	6.11	Proteus phage vB_PmiM	Baseplate wedge subunit (AKA61998.1)	800E-101 (76)	Phage_gp53 (PF11246.5)
144/+	72377	74113	578	63.45	4.74	Proteus phage vB_PmiM	Baseplate hub subunit and tail lysozyme (AKA61999.1)	0.0 (55)	Phage_lysozyme (pfam00959); Gp5_OB (PF06714.8)
145/+	74158	74592	144	16.42	4.49	E. coli phage vB_EcoM_JS09	Hypothetical protein (YP_009037343.1)	200E-38 (44)	-
146/+	74592	74885	97	10.26	8.51	Klebsiella phage JD18	Hypothetical protein (AKY02017.1)	200E-44 (74)	PAAR_motif (PF05488.10)
147/+	74882	76789	635	72.37	4.57	Proteus phage vB_PmiM	Baseplate wedge subunit (AKA62001.1)	0.0 (72)	-
148/+	76789	79932	1047	121.22	5.31	Proteus phage vB_PmiM	Baseplate wedge subunit (AKA62002.1)	0.0 (69)	-
149/+	79929	80930	333	38.63	4.68	Proteus phage vB_PmiM	Baseplate wedge tail fiber connector (AKA62003.1)	0.0 (77)	Phage-Gp8 (PF09215.7)
150/+	80988	81842	284	31.34	6.17	Proteus phage vB_PmiM	Baseplate wedge subunit and tail pin (AKA62004.1)	500E-135 (67)	T4_gp9_10 (PF09215.7)
151/+	81839	83644	601	67.11	4.27	Citrobacter phage Moon	Baseplate wedge subunit and tail pin (YP_009146609.1)	0.0 (48)	T4_gp9_10 (PF09215.7)
152/+	83644	84279	211	23.26	4.31	Proteus phage vB_PmiM	Baseplate wedge subunit and tail pin (AKA62007.1)	300E-45 (45)	GP11 (PF08677.7)
153/+	84279	85595	438	47.61	8.02	Proteus phage vB_PmiM	Short tail fibers (AKA62008.1)	300E-133 (49)	-
154/+	85607	86962	451	49.51	4.48	Proteus phage vB_PmiM	Fibritin neck whiskers (AKA62009.1)	900E-125 (47)	Fibritin_C (PF07921.9)
155/+	86923	87348	141	15.78	4.45	Proteus phage vB_PmiM	Fibritin neck whiskers (AKA62009.1)	400E-05 (28)	-
156/+	87382	88308	308	34.19	4.41	Proteus phage vB_PmiM	Neck (AKA62010.1)	400E-172 (75)	-
157/+	88313	89053	246	28.86	4.18	Proteus phage vB_PmiM	Neck (AKA62011.1)	200E-126 (72)	T4_neck-protein (PF11649.5)
158/+	89063	89884	273	32.38	4.87	Proteus phage vB_PmiM	Tail sheath stabilization (AKA62012.1)	800E-134 (68)	T4-gp15_tss (PF16724.2)
159/+	89887	90357	156	17.61	4.48	Proteus phage vB_PmiM	Terminase small subunit (AKA62013.1)	800E-77 (76)	DNA_Packaging (PF11053.5)
160/+	90338	92137	599	68.78	4.81	Proteus phage vB_PmiM	Terminase large subunit (AKA62014.1)	0.0 (84)	Terminase_6 (PF03237.12)
161/+	92160	94148	662	72.62	5.44	Proteus phage vB_PmiM	Tail sheath (AKA62015.1)	0.0 (76)	Phage_sheath_1 (PF04984.11)
162/+	94187	94678	163	18.57	4.82	Proteus phage vB_PmiM	Tail tube (AKA62016.1)	300E-97 (79)	Phage_T4_gp19 (PF06841.9)
163/+	94728	96287	519	60.3	5.27	Proteus phage vB_PmiM	Portal vertex of head (AKA62017.1)	0.0 (68)	Peptidase_S80 (PF07230.8)
164/+	96288	96503	71	8.24	4.26	Salmonella phage STP4-a	Prohead core (YP_009126365.1)	200E-08 (48)	-
165/+	96506	96925	139	16.19	11.14	Proteus phage vB_PmiM	Prohead core (AKA62019.1)	300E-51 (70)	-
166/+	96927	97556	209	23.13	5.16	Proteus phage vB_PmiM	Prohead core scaffold and Protease (AKA62020.1)	200E-111 (79)	Peptidase_S77 (PF03420.10)

167/+	97589	98395	268	30.36	4.15	Proteus phage vB_PmiM	Prohead core scaffold (AKA62021.1)	300E-77 (56)	-
168/+	98410	99960	516	56.01	4.86	Proteus phage vB_PmiM	Major capsid (AKA62022.1)	0.0 (81)	Gp23 (PF07068.8)
169/-	100051	101298	415	46.16	4.47	Proteus phage vB_PmiM	Head vertex (AKA62023.1)	0.0 (78)	Gp23 (PF07068.8)
170/-	101364	101774	136	15.9	7.50	Proteus phage vB_PmiM	Hypothetical protein (AKA62024.1)	400E-42 (54)	-
171/-	101905	102153	82	9.63	10.99	Proteus phage vB_PmiM	Hypothetical protein (WP_046099516.1)	300E-07 (33)	1 TMD
172/-	102155	102436	93	10.77	5.03	-	-	-	-
173/-	102423	103418	331	38.14	5.38	Proteus phage vB_PmiM	RNA ligase 2 (AKA62027.1)	300E-131 (57)	RNA_ligase (PF09414.7)
174/-	103411	103770	119	14.17	5.16	Proteus phage vB_PmiM	Hypothetical protein (AKA62028.1)	800E-10 (34)	-
175/-	103760	104020	86	10.24	5.15	-	-	-	-
176/-	104017	104661	214	25.16	7.82	Proteus phage vB_PmiM	Hypothetical protein (AKA62028.1)	700E-07 (34)	-
177/-	104698	104889	63	7.49	10.68	Proteus phage vB_PmiM	Hypothetical protein (AKA62030.1)	200E-19 (60)	DUF2774 (PF11242.5)
178/-	104882	105331	149	16.85	8.94	Desulfotomaculum kuznetsovii	Hypothetical protein (WP_013822025.1)	200E-21 (34)	-
179/-	105328	105783	151	17.44	4.15	Proteus phage vB_PmiM	Hypothetical protein (AKA62034.1)	100E-32 (46)	-
180/-	105770	105976	68	7.89	4.23	-	-	-	-
181/-	105973	106293	106	12.14	5.84	-	-	-	-
182/-	106290	107048	252	29.99	4.41	-	-	-	-
183/-	107059	107259	66	7.76	3.97	-	-	-	-
184/-	107261	107599	112	13.48	5.67	-	-	-	-
185/-	107754	108023	89	10.7	6.09	-	-	-	-
186/-	108026	108214	62	7.36	4.70	Proteus phage vB_PmiM	Hypothetical protein (AKA62113.1)	800E-11 (53)	-
187/-	108302	109216	304	33.42	4.63	Proteus phage vB_PmiM	Baseplate tail tube initiator (AKA62038.1)	700E-174 (79)	Phage_T4_gp19 (PF06841.9)
188/-	109216	110262	348	38.28	6.81	Proteus phage vB_PmiM	Baseplate tail tube cap (AKA62039.1)	0.0 (75)	T4_tail_cap (PF11091.5)
189/-	110262	111962	566	62.73	4.61	Proteus phage vB_PmiM	Baseplate hub subunit and tail length determinator (AKA62040.1)	300E-119 (41)	1 TMD
190/-	111946	112425	159	18.18	5.68	Proteus phage vB_PmiM	Baseplate hub distal subunit (AKA62041.1)	300E-62 (59)	Phage_hub_GP28 (PF11110.5)
191/-	112406	113572	388	44.55	4.84	Proteus phage vB_PmiM	Baseplate hub subunit (AKA62042.1)	0.0 (68)	Phage-tail_1 (PF09097.7); Phage-tail_2 (PF09096.7)
192/+	113572	114333	253	29.97	5.14	Proteus phage vB_PmiM	Baseplate hub assembly (AKA62043.1)	100E-84 (53)	1 TMD; T4_baseplate (PF12322.5)
193/+	114380	114979	199	23.04	4.47	Proteus phage vB_PmiM	Baseplate hub subunit (AKA62044.1)	100E-40 (41)	T4_baseplate (PF12322.5)
194/+	114972	115358	128	14.38	4.71	Proteus phage vB_PmiM	Baseplate wedge subunit (AKA62045.1)	900E-56 (66)	Gene 25-like lysozyme (pfam04965)
195/+	115565	115975	136	15.74	8.63	Proteus phage vB_PmiM	recombination repair and ssDNA binding protein (AKA62046.1)	300E-65 (71)	UvsY (PF11056.5)

196/+	115975	116187	70	8.25	4.73	-	-	-	-
197/-	116188	116349	53	6.12	4.01	Proteus phage vB_PmiM	Hypothetical protein (AKA62047.1)	100E-06 (48)	DUF2685 (PF10886.5)
198/-	116341	116568	75	8.66	4.06	Edwardsiella phage PEi20	RNA-DNA and DNA-DNA helicase ATPase (BAQ22851.1)	300E-17 (56)	UvsW (PF11637.5)
199/+	116579	118072	497	57.62	9.43	Enterobacteria phage vB_EcoM_VR5	Helicase (AIZ01970.1)	0.0 (69)	ResIII (PF04851.12); Helicase_C (PF00271.28)
200/+	118131	118853	240	27.48	4.28	Proteus phage vB_PmiM	Inhibitor of prohead protéase (AKA62050.1)	500E-83 (55)	-
201/-	118865	119950	361	39.71	4.51	Proteus phage vB_PmiM	Head outer capsid (AKA62051.1)	100E-176 (68)	-
202/-	119984	120595	203	23.64	4.84	-	-	-	-
203/-	120595	120939	114	13	4.03	-	-	-	-
204/-	120941	121228	95	10.84	4.44	-	-	-	-
205/-	121291	123345	684	77.21	5.27	Proteus phage vB_PmiM	RNA polymerase ADP-ribosylase (AKA62054.1)	0.0 (49)	ADPrib_exo_Tox (PF03496.11)
206/-	123405	124874	489	55.74	4.78	Proteus phage vB_PmiM	DNA ligase (AKA62057.1)	0.0 861)	DNA_ligase_A_M (PF01068.18)
207/-	124875	125513	212	24.41	5.89	Proteus phage vB_PmiM	Hypothetical protein (AKA62058.1)	300E-126 (84)	-
208/-	125510	125965	151	17.27	9.67	Proteus phage vB_PmiM	Hypothetical protein (AKA62061.1)	500E-47 (55)	Phage_30_3 (PF08010.8)
209/-	125966	126418	150	17.46	5.10	-	-	-	-
210/-	126415	126813	132	15.53	4.44	-	-	-	-
211/-	126824	127246	140	15.97	4.19	-	-	-	-
212/-	127246	127509	87	10.14	4.65	-	-	-	-
213/-	127559	127915	118	13.58	7.05	Citrobacter phage Merlin	Hypothetical protein (AKU43875.1)	100E-25 (46)	Phage_T4_Gp30_7 (PF06919.8)
214/-	127986	128351	121	14.22	4.67	Streptomyces ahygroscopicus	Hypothetical protein (WP_044370027.1)	600E-38 (57)	Phage_30_8 (PF06019.8)
215/-	128452	128688	78	9.25	4.78	Proteus phage vB_PmiM	Hypothetical protein (AKA62063.1)	300E-37 (79)	-
216/-	128804	129049	81	9.32	9.93	Proteus phage vB_PmiM	rIII lysis inhibition accessory protein rapid lysis phenotype (AKA62065.1)	200E-40 (81)	-
217/-	129108	129428	106	11.49	6.53	Proteus phage vB_PmiM	Head assembly cochaperone with GroEL (AKA62066.1)	500E-42 (67)	Cpn10 (PF00166.18)
218/-	129428	129733	101	11.55	5.76	Klebsiella phage JD18	Hypothetical protein (AKY02076.1)	200E-17 (43)	DUF2693 (PF10902.5)
219/-	129730	130281	183	20.2	6.77	Enterobacteria phage vB_EcoM_VR5	Deoxycytidylate deaminase (AIZ02003.1)	900E-83 (66)	dCMP_cyt_deam_1 (PF00383.20)
220/-	130281	130568	95	10.95	4.36	Acinetobacter phage 133	Hypothetical protein (YP_004300794.1)	200E-04 (42)	-
221/-	130568	130843	91	10.39	4.14	-	-	-	-
222/-	130843	131052	69	7.51	4.46	-	-	-	-
223/-	131049	131246	65	7.39	5.75	-	-	-	-
224/-	131249	131494	81	9.41	3.86	-	-	-	-

225/-	131521	132237	238	26.96	10.19	Acinetobacter phage 133	Seg-like homing endonuclease (YP_004300718.1)	900E-28 (59)	-
226/-	132294	133190	298	34.97	7.98	Proteus phage vB_PmiM	3'-phosphatase 5'-polynucleotide kinase (AKA62075.1)	200E-124 (60)	AAA_33 (PF13671.3)
227/-	133187	133681	164	18.75	4.89	Proteus phage vB_PmiM	Hypothetical protein (AKA62077.1)	100E-18 (35)	-
228/-	133748	134032	94	11.02	8.46	Enterobacteria phage RB3	Outer membrane lipoprotein (YP_009098605.1)	100E-27 (49)	1 SP
229/-	134029	134379	116	13.3	9.97	Edwardsiella phage PEi26	Hypothetical protein (BAQ23193.1)	100E-06 (35)	1 TMD; 1 SP
230/-	134367	134867	166	19.35	8.57	Proteus phage vB_PmiM	Inhibitor of host transcription (AKA62080.1)	100E-54 (55)	-
231/-	134927	136021	364	42.48	4.81	Proteus phage vB_PmiM	RNA ligase 1 and tail fiber attachment catalyst (AKA62081.1)	500E-147 (58)	RNA_lig_T4_1 (PF09511.7)
232/-	136008	136139	43	5.11	9.32	Enterobacteria phage CC31	endonuclease II (YP_004010090.1)	700E-10 (90)	-
233/-	136485	137672	395	45.94	4.61	Proteus phage vB_PmiM	Aerobic NDP reductase small subunit (AKA62083.1)	0.0 (66)	Ribonuc_red_sm (PF00268.18)
234/-	137659	138405	248	28.51	10.25	Enterobacteria phage RB3	Homing endonuclease (YP_009098612.1)	100E-114 (67)	NUMOD3 (PF07460.8); NUMOD1 (PF07453.10)
235/-	138406	140640	744	84.8	5.73	Proteus phage vB_PmiM	Aerobic NDP reductase large subunit (AKA62085.1)	0.0 (72)	ATP-cone (PF03477.13); Ribonuc_red_lgN (PF00317.18); Ribonuc_red_lgC (PF02867.12)
236/-	140642	140875	77	9.21	8.06	Enterobacteria phage vB_EcoM_VR20	Hypothetical protein (AIZ02300.1)	400E-30 (76)	DUF4326 (PF14216.3)
237/-	141002	141268	88	10.5	5.15	-	-	-	-
238/-	141277	141555	92	10.64	5.90	-	-	-	-
239/-	141647	141952	101	11.47	3.72	-	-	-	-
240/-	142020	142859	279	32.28	6.31	Proteus phage vB_PmiM	Thymidylate synthase (AKA62089.1)	100E-148 (72)	Thymidylat_synt (PF00303.16)
241/-	142860	143417	185	20.66	4.65	Acinetobacter phage Acj61	Hypothetical protein (YP_004009740.1)	800E-28 (38)	-
242/-	143404	143649	81	9.24	4.48	Enterobacteria phage phi92	Hypothetical protein (YP_009012530.1)	200E-24 (56)	-
243/-	143652	143864	70	7.74	4.20	-	-	-	-
244/-	143875	144297	140	16.42	9.29	Proteus phage vB_PmiM	Hypothetical protein (AKA62091.1)	300E-11 (31)	-
245/-	144284	144883	199	22.43	4.63	Proteus phage vB_PmiM	Dihydrofolate reductase (WP_044369981.1)	600E-21 (36)	DHFR_1 (PF00186.16)
246/-	144973	145860	295	32.67	4.47	Proteus phage vB_PmiM	ssDNA-binding protein (AKA62094.1)	900E-148 (71)	gp32 (PF08804.7)
247/-	145896	146546	216	25.7	9.63	Proteus phage vB_PmiM	DNA helicase loading protein (AKA62095.1)	400E-116 (77)	T4_Gp59_N (PF08993.7); T4_Gp59_C (PF08994.7)
248/-	146543	146815	90	10.42	4.36	Streptomyces ahygroscopicus	late promoter transcription accessory protein (WP_016031138.1)	700E-27 (55)	Trans_coact (PF16805.2)
249/-	146790	147059	89	10.36	4.63	Proteus phage vB_PmiM	dsDNA binding protein (AKA62097.1)	800E-41 (75)	Phage_DsbA (PF11126.5)
250/+	147071	147988	305	35.75	6.54	Proteus phage vB_PmiM	RNase H (AKA62098.1)	200E-139 (65)	5_3_exonuc_N (PF02739.13); RNaseH_C (PF09293.7)
251/+	148037	151687	1216	134.62	4.92	Proteus phage vB_PmiM	Long tail fiber proximal subunit (AKA62099.1)	0.0 (60)	-

252/+	151689	152825	378	43.15	4.64	Proteus phage vB_PmiM	Long tail fiber proximal connector (AKA62100.1)	300E-102 (44)	-
253/+	153014	153496	160	17.3	4.19	Proteus phage vB_PmiM	Long tail fiber distal connector (AKA62101.1)	200E-24 (40)	Phage_T4_gp36 (PF03903.10)
254/+	153496	155202	568	61.7	8.66	Proteus phage vB_PmiM	Long tail fiber distal subunit (AKA62102.1)	200E-22 (46)	-
255/-	155211	155834	207	24.06	6.12	Proteus phage vB_PmiM	Holin lysis mediator (AKA62104.1)	100E-90 (64)	Phage_holin_T (pfam11031) ; 1 TMD
256/-	155835	156026	63	7.59	10.52	Yersinia phage phiR1-RT	Hypothetical protein (YP_007235932.1)	100E-19 (67)	-
257/-	155998	156474	158	18.56	6.12	Proteus phage vB_PmiM	Hypothetical protein (AKA62105.1)	200E-08 (27)	-
258/-	156462	156773	103	12	4.54	Proteus phage vB_PmiM	Hypothetical protein (AKA62106.1)	400E-11 (42)	-
259/-	156773	157141	122	14.03	4.07	Proteus phage vB_PmiM	Hypothetical protein (AKA62107.1)	500E-11 (37)	-
260/-	157148	157450	100	11.52	8.02	Enterobacteria phage vB_KleM-RaK2	Hypothetical protein (YP_007007436.1)	100E-04 (56)	-
261/-	157463	157657	64	7.75	4.36	-	-	-	-
262/-	157665	157943	92	10.69	5.63	-	-	-	-
263/-	157943	158116	57	6.71	10.38	-	-	-	-
264/-	158118	158378	86	10.07	4.63	Proteus phage vB_PmiM	Anti-sigma 70 protein (AKA62109.1)	400E-21 (50)	AsiA (PF09010.7)
265/-	158375	158950	191	21.17	4.81	Proteus phage vB_PmiM	Activator of middle transcription (AKA62111.1)	500E-37 (37)	MotCF (PF09158.7)
266/-	159046	160407	453	51.72	7.36	Proteus phage vB_PmiM	DNA topoisomerase (AKA62115.1)	0.0 (63)	DNA_topoisoIV (PF00521.17)
267/-	160445	160897	150	17.41	9.78	Proteus phage vB_PmiM	Nucleoid disruption protein (AKA62116.1)	900E-25 (44)	Phage_T4_Ndd (PF06591.8)
268/-	160897	161445	182	21.09	6.68	Acinetobacter phage 133	DenB DNA endonuclease IV (YP_004300837.1)	100E-31 (40)	-
269/-	161492	161986	164	19.08	10.20	Enterobacteria phage vB_EcoM-VR7	Hypothetical protein (YP_004063973.1)	900E-80 (69)	AP2 (PF00847.17)
270/-	161986	163197	403	45.68	4.64	Edwardsiella phage PEi26	rIIB protector from prophage-induced early lysis (BAQ23251.1)	500E-107 (63)	-

TMD – transmembrane domain; SP – Signal peptide

Table S3. Features of MP2 predicted CDSs. For each CDS the transcription start and stop position, and the coding strand is given. At protein level the corresponding gene product size molecular weight and pI as well as the homolog predicted function homology, values and motifs are shown.

			Predicted protein						
ORF (+/-)	Start (bp)	Stop (bp)	Size (aa)	MW (Kda)	pI	Best species hit	Putative function (accession no.)	E-value (% identify)	Motifs
1/+	611	769	52	5.89	10.00	Citrobacter phage CR8	Hypothetical protein (YP_009004161.1)	1e-08 (56)	-
2/+	858	1,106	82	9.48	4.42	-	-	-	-
3/+	1,194	1,574	126	14.65	3.52	Morganella phage MmP1	Hypothetical protein (YP_002048671.1)	5e-32 (64)	ocr (PF08684.7)
4/+	1,567	1,770	67	7.68	4.14	-	-	-	-
5/+	1,831	2,022	63	7.20	4.07	-	-	-	4 TMD
6/+	2,038	2,334	98	11.55	10.46	-	-	-	-
7/+	2,336	2,611	91	10.71	5.58	-	-	-	-
8/+	2,604	3,119	171	19.10	6.11	-	-	-	-
9/+	3,246	3,464	72	8.28	4.05	-	-	-	-
10/+	3,554	6,205	883	100.17	7.24	Morganella phage MmP1	RNA Polymerase (YP_003324580.1)	0 (92)	RPOL_N (PF14700.3); RNA_pol (PF00940.169
11/+	6,538	6,699	53	6.43	11.04	Morganella phage MmP1	Hypothetical protein (YP_002048631.1)	2e-09 (96)	-
12/+	6,704	6,943	79	9.68	8.48	-	-	-	-
13/+	6,948	7,979	343	38.84	4.99	Morganella phage MmP1	DNA Ligase (YP_002048633.1)	0 (98)	DNA_ligase_A_M (PF01068.18)
13A/+	7,981	8,154	57	6,34	9,38	-	-	-	2 TMD, 1 SP
13B/+	8,181	8,360	59	6,70	7,91	-	-	-	2 TMD
13C/+	8,435	8,542	35	4.01	6.50	-	-	-	1 TMD
14/+	8,599	8,874	91	10.63	11.87	Morganella phage MmP1	Hypothetical protein (YP_002048636.1)	7e-44 (77)	-
15/+	8,876	9,460	194	21.72	8.04	Morganella phage MmP1	Hypothetical protein (YP_002048637.1)	3e-48 (53)	DUF3310 (PF11753.5)
16/+	9,438	9,608	56	6.79	4.44	Morganella phage MmP1	Hypothetical protein (YP_002048638.1)	1e-13 (70)	-
17/+	9,595	9,804	69	7.49	4.27	Morganella phage MmP1	RNA polymerase inhibitor (YP_002048639.1)	7e-32 (77)	RNA_pol_inhib (PF16857.2)
18/+	9,855	10,574	239	26.47	4.24	Morganella phage MmP1	single-stranded DNA-binding (YP_002048640.1)	2e-89 (87)	-
19/+	10,574	11,017	147	17.23	9.98	Morganella phage MmP1	Endonuclease I (YP_002048641.1)	5e-94 (93)	Phage_endo_I (PF05367.8)
20/+	11,039	11,263	74	8.31	8.75	-	-	-	-
21/+	11,260	11,721	153	17.37	9.63	Morganella phage MmP1	Endolysin (YP_002048642.1)	8e-102 (93)	Amidase_2 (PF01510)
22/+	11,798	12,034	78	8.54	4.14	Morganella phage MmP1	Hypothetical protein (YP_002048643.1)	8e-10 (54)	-
23/+	12,064	13,803	579	64.08	4.96	Morganella phage MmP1	DNA Primase/Helicase (YP_002048644.1)	0 (86)	Prim_Zn_Ribbon (PF08273.9); Toprim_2 (PF13155.3); DnaB_C (PF03796.12)
24/+	13,763	13,885	40	4.76	8.49	-	-	-	-
25/+	13,898	14,110	70	7.83	10.62	Morganella phage MmP1	Hypothetical protein (YP_002048645.1)	3e-24 (93)	-
25A/+	14,120	14,299	59	6.98	9.57	-	-	-	-

26/+	14,296	14,526	76	9.24	8.98	Morganella phage MmP1	hypothetical protein (YP_002048646.1)	6e-25 (76)	-
27/+	14,604	16,712	702	79.76	6.85	Morganella phage MmP1	DNA polymerase (YP_002048647.1)	0 (89)	DNA_pol_A (PF00476.17)
28/+	16,737	17,063	108	12.76	6.62	Morganella phage MmP1	Hypothetical protein (YP_002048648.1)	6e-17 (54)	-
29/+	17,068	17,355	95	10.52	5.12	-	-	-	-
30/+	17,376	17,585	69	7.56	8.48	Morganella phage MmP1	Hypothetical protein (YP_002048650.1)	3e-40 (93)	-
31/+	17,591	17,803	70	8.05	5.06	Morganella phage MmP1	Hypothetical protein (YP_002048651.1)	6e-38 (86)	-
32/+	17,794	18,711	305	35.06	5.05	Morganella phage MmP1	Exonuclease (YP_002048652.1)	0 (90)	5_3_exonuc_N (PF02739.13)
33/+	18,854	19,102	82	9.36	5.10	Morganella phage MmP1	Hypothetical protein (ACF42032.2)	3e-17 (74)	DUF2717 (PF10911.5)
34/+	19,121	19,357	78	8.44	10.54	Morganella phage MmP1	Hypothetical protein (YP_002048654.1)	3e-23 (70)	-
35/+	19,381	19,614	77	7.93	10.11	Escherichia phage CICC 80001	Host Range Protein (YP_009152490.1)	2e-04 (46)	VirionAssem_T7 (PF11653.5)
36/+	19,631	21,262	543	59.35	4.35	Morganella phage MmP1	Head-to-tail joining protein (YP_002048655.1)	0 (92)	Head-tail_con (PF12236.5)
37/+	21,386	22,354	322	35.43	4.11	Morganella phage MmP1	Capsid assembly protein (YP_002048656.1)	0 (80)	Phage_T7_Capsid (PF05396.8)
38/+	22,485	23,516	343	36.23	6.18	Morganella phage MmP1	Major capsid protein (YP_002048657.1)	0 (89)	-
39/+	23,516	23,647	43	4.69	6.76	Morganella phage MmP1	Hypothetical protein (YP_002048658.1)	7e-05 (80)	-
40/+	23,718	24,311	197	22.09	4.22	Morganella phage MmP1	Tail tubular protein A (YP_002048659.1)	6e-99 (86)	-
41/+	24,331	26,790	819	90.77	5.74	Morganella phage MmP1	Tail tubular protein B (YP_002048660.1)	0 (88)	-
42/+	26,867	27,280	137	15.72	5.18	Morganella phage MmP1	Internal virion protein A (YP_002048661.1)	5e-84 (87)	DUF2833 (PF11090.5)
43/+	27,280	27,858	192	20.41	10.07	Morganella phage MmP1	Internal virion protein B (YP_002048662.1)	1e-87 (84)	-
44/+	27,858	30,062	734	82.57	4.67	Morganella phage MmP1	Internal virion protein C (YP_002048663.1)	0 (76)	-
45/+	30,081	34,010	1309	143.87	6.91	Morganella phage MmP1	Internal virion protein D (YP_002048664.1)	0 (70)	STL (PF01464); 2 TMD
46/+	34,101	35,819	572	63.26	8.41	Morganella phage MmP1	Tail fiber protein (YP_002048665.1)	0 (72)	Phage_holin_2_2 (PF10746); 1 TMD
47/+	35,867	36,091	74	7.94	6.67	Morganella phage MmP1	Holin (YP_002048666.1)	8e-44 (100)	Phage_lysis (PF03245)
48/+	36,075	36,347	90	10.22	4.84	Morganella phage MmP1	DNA packaging small subunit (YP_002048667.1)	2e-48 (87)	DNA_Packaging_2 (PF11123.5)
49/+	36,452	36,898	148	16.57	9.29	Morganella phage MmP1	Rz-like lysis protein (YP_002048669.1)	2e-99 (99)	Phage_lysis (PF03245.10)
50/+	36,912	38,672	586	66.55	6.09	Morganella phage MmP1	DNA packaging protein B (YP_002048670.1)	0 (92)	1 TMD
50A/+	38,946	39,056	36	4,05	10,45	-	-	-	-

TMD – transmembrane domain; SP – Signal peptide

Figure S1. Cytotoxicity assay of MP1 and MP2 on mammalian cells. The cytotoxic effect was evaluated by incubating SM buffer (as a negative control), MP1 or MP2 with a end concentration of 10^8 PFUs/mL, with a cell line A549 (ATCC CCL-185) for 24 h. The amount of soluble formazan produced by cellular reduction of MTS was measured by reading the absorbance at 490 nm, after addition of the CellTiter 96 Aqueous One Solution Reagent. The experiment was performed in triplicate.

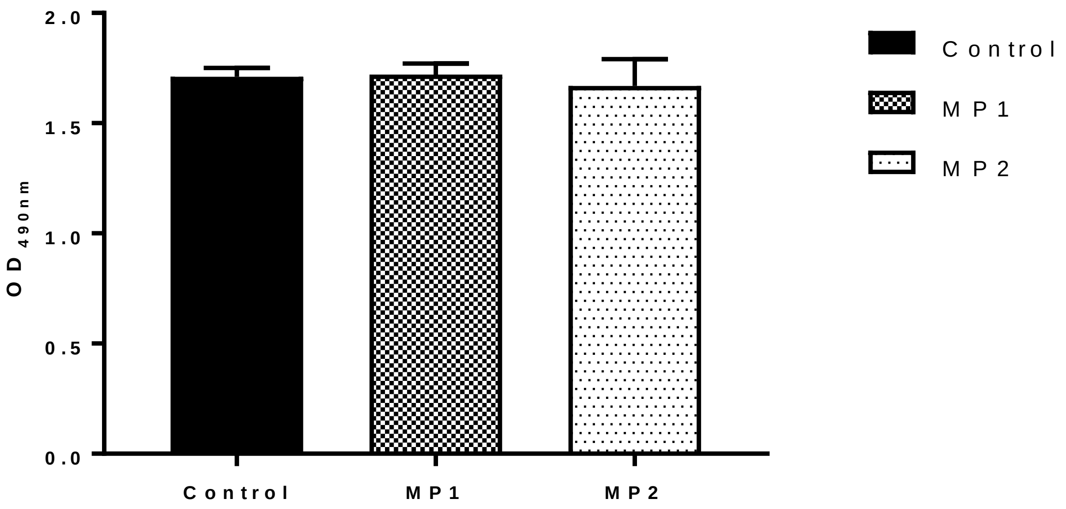

Figure S2. Nucleotide dot plot analysis of *Morganella* phage genomes. Nucleic acid comparisons of A) MP1 or B) MP2 genomes were compared to *Tevenvirinae* and *Autographivirinae* like phages, respectively. Fasta files of whole genome sequences were downloaded from GenBank and relationships were assigned using Gepard, which are indicated by the diagonal lines.

A)

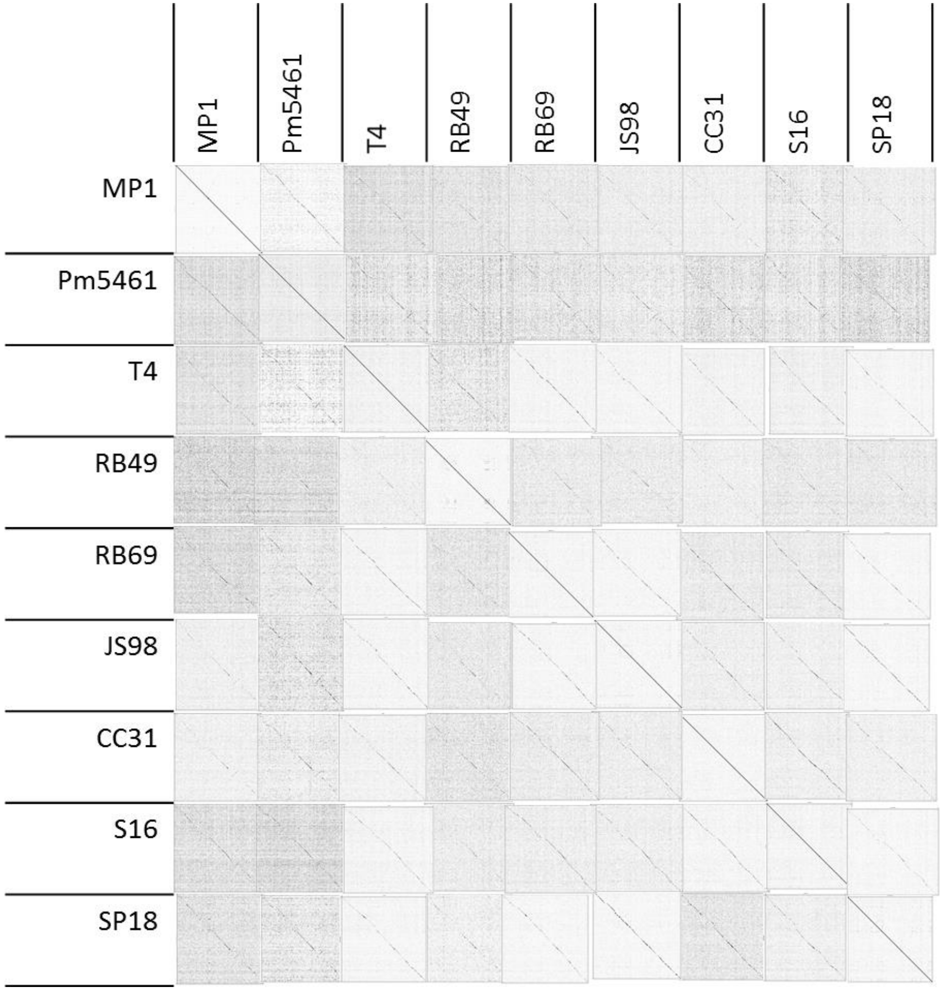

B)

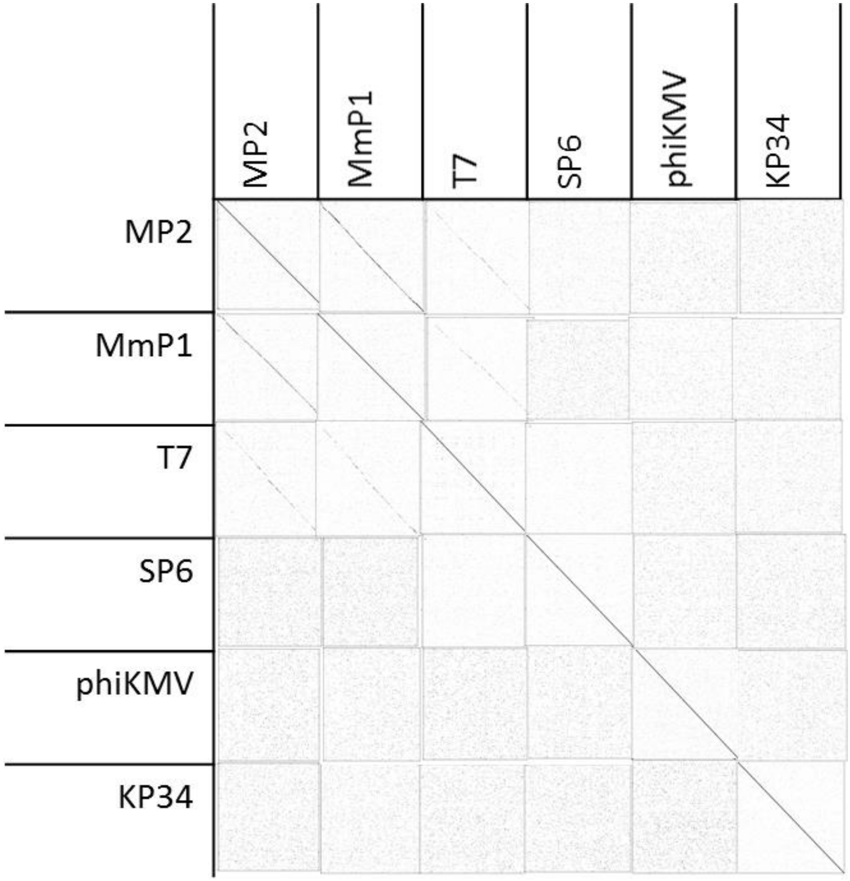

Supplement: Supplementary Information [file srep46157-s1.pdf]
